# A novel *Porphyromonas gingivalis* enzyme: An atypical dipeptidyl peptidase III with an ARM repeat domain

**DOI:** 10.1371/journal.pone.0188915

**Published:** 2017-11-30

**Authors:** Altijana Hromić-Jahjefendić, Nina Jajčanin Jozić, Saša Kazazić, Marina Grabar Branilović, Zrinka Karačić, Jörg H. Schrittwieser, Krishna Mohan Padmanabha Das, Marko Tomin, Monika Oberer, Karl Gruber, Marija Abramić, Sanja Tomić

**Affiliations:** 1 Institute of Molecular Biosciences, University of Graz, Graz, Austria; 2 Sir William Dunn School of Pathology, University of Oxford, Oxford, United Kingdom; 3 Division of Physical Chemistry, Ruđer Bošković Institute, Zagreb, Croatia; 4 Division of Organic Chemistry and Biochemistry, Ruđer Bošković Institute, Zagreb, Croatia; 5 Institute of Chemistry, University of Graz, Graz, Austria; Medical University of South Carolina, UNITED STATES

## Abstract

*Porphyromonas gingivalis*, an asaccharolytic Gram-negative oral anaerobe, is a major pathogen associated with adult periodontitis, a chronic infective disease that a significant percentage of the human population suffers from. It preferentially utilizes dipeptides as its carbon source, suggesting the importance of dipeptidyl peptidase (DPP) types of enzyme for its growth. Until now DPP IV, DPP5, 7 and 11 have been extensively investigated. Here, we report the characterization of DPP III using molecular biology, biochemical, biophysical and computational chemistry methods. In addition to the expected evolutionarily conserved regions of all DPP III family members, *Pg*DPP III possesses a C-terminal extension containing an Armadillo (ARM) type fold similar to the AlkD family of bacterial DNA glycosylases, implicating it in alkylation repair functions. However, complementation assays in a DNA repair-deficient *Escherichia coli* strain indicated the absence of alkylation repair function for *Pg*DPP III. Biochemical analyses of recombinant *Pg*DPP III revealed activity similar to that of DPP III from *Bacteroides thetaiotaomicron*, and in the range between activities of human and yeast counterparts. However, the catalytic efficiency of the separately expressed DPP III domain is ~1000-fold weaker. The structure and dynamics of the ligand-free enzyme and its complex with two different diarginyl arylamide substrates was investigated using small angle X-ray scattering, homology modeling, MD simulations and hydrogen/deuterium exchange (HDX). The correlation between the experimental HDX and MD data improved with simulation time, suggesting that the DPP III domain adopts a semi-closed or closed form in solution, similar to that reported for human DPP III. The obtained results reveal an atypical DPP III with increased structural complexity: its superhelical C-terminal domain contributes to peptidase activity and influences DPP III interdomain dynamics. Overall, this research reveals multifunctionality of *Pg*DPP III and opens direction for future research of DPP III family proteins.

## Introduction

*Porphyromonas gingivalis* is a major pathogen that can be found in the oral cavity of mammals and represents a major cause of chronic periodontitis [1] which affects a significant percentage of the human population [2]. This Gram-negative oral anaerobe colonizes the subgingival region by adherence to adsorbed salivary molecules, matrix proteins, epithelial cells and bacteria that are already established as a biofilm on tooth and epithelial surfaces [3,4]. Although periodontal disease is localized to the tissues surrounding the tooth, accumulating evidence indicates that infection of *P*. *gingivalis* in the bloodstream contributes to systemic diseases such as endocarditis and pulmonary infections [5–7]. Other studies show periodontal disease increases the risk of developing colorectal cancer [8], lung cancer [9] and pancreatic cancer [10]. Periodontal disease represents also a risk factor for non-Hodgkin lymphoma [11]. There is also a demonstrated link between diagnosed periodontitis and the risk of ischemic stroke [12].

*P*. *gingivalis* is an asaccharolytic organism that has growth requirements for hemin as a source of iron and peptides as a source of carbon and nitrogen. Therefore it possesses a complex cell surface-associated proteolytic system comprising several unique peptidases [13]. Among them, the best-characterized enzymes are gingipains R and K, arginine- and lysine-specific cysteine endopeptidases, which are major *P*. *gingivalis* virulence factors [14]. Gingipains are present on the cell surface, but also in extracellular membrane vesicles and in culture supernatants of *P*. *gingivalis*. These proteases are able to degrade many human proteins: constituents of connective tissue, cell surface proteins and receptors, cytokines and plasma proteins including components of the coagulation and complement cascades, heme- and iron-binding proteins, immunoglobulins, and proteinase inhibitors. Through their multiple pathogenic effects, gingipains are implicated in most phases of periodontal disease pathogenesis, from adherence and colonization to nutrient supply and attenuation of host defenses [15]. Additionally, dipeptidyl peptidase IV (DPP IV) is important for *P*. *gingivalis* pathogenicity, as disruption of the gene coding for this enzyme reduces its virulence. DPP IV is a serine protease that cleaves X-Pro and X-Ala dipeptides at the N-terminus of (poly)peptides [16], *P*. *gingivalis* DPP IV is reported to contribute to degradation of connective tissue by promoting the activity of host-derived collagenase (MMP-1) and gelatinase (MMP-2) [17]. Furthermore, this enzyme also mediates the adhesion of *P*. *gingivalis* to fibronectin.

The availability of the complete genome sequence of *P*. *gingivalis* W83 facilitates detailed investigation of this pathogen [18]. The *P*. *gingivalis* genome encodes a total of 42 peptidases [18]. In addition to the previously described gingipains that are known to degrade host proteins, 36 peptidases are newly identified. They might be involved in further processing of protein fragments to smaller peptides and amino acids.

Using a sequence similarity search and multiple sequence alignments (S1 Fig), we found a member of the metallopeptidase family M49 (DPP III family) encoded in the *P*. *gingivalis* W83 genome (ORF name: PG_0317), under the UniProtKB entry Q7MX92_PORGI.

DPP III in eukaryotes has been extensively investigated. It is a broadly distributed, predominantly cytosolic zinc-metallopeptidase that cleaves dipeptides from the N-termini of various peptides consisting of three to ten amino acids [19,20]. Participation in the final steps of protein catabolism is considered to be one of the physiological roles of eukaryotic DPP III [19,20]. The mammalian enzyme is additionally involved in cellular defense against oxidative stress, as an activator in the KEAP1-Nrf2 signaling pathway [21]. This is exerted through protein-protein interaction and does not require the catalytic activity of DPP III.

Until now, only one bacterial DPP III has been characterized biochemically, the enzyme from the gut symbiont *Bacteroides thetaiotaomicron* (*Bt*DPP III) [22,23]. Kinetic and structural studies revealed similarities but also differences between the properties of *Bt*DPP III and its eukaryotic DPP III homologs [24–26].

According to a bioinformatic analysis of the predicted amino acid sequence, peptidase M49 from *P*. *gingivalis* W83 contains the hexapeptide HEXXXH active site motif, and four additional evolutionarily conserved regions of the DPP III family [27], all of which are essential for the catalytic function of DPP III. Therefore, it is reasonable to assume that *P*. *gingivalis* M49 peptidase (DPP III) would cleave N-terminal dipeptides from its substrates. The findings of Takahashi and Sato [28, 29] clearly indicate that *P*. *gingivalis* uses dipeptides preferentially as its sole source of carbon, suggesting the importance of dipeptidyl peptidase type of enzymes for the growth of this asaccharolytic bacterium. This indicates that peptidase M49 might be an important hydrolase for the survival of the bacterium *P*. *gingivalis* W83. This motivated us to characterize this protein using a combination of experimental and computational approaches.

## Materials and methods

### Cloning

The gene encoding full-length *Pg*DPP III consisting of 886 aa (*PG_0317*, 2661 bp) was obtained by PCR using genomic DNA of *P*. *gingivalis* strain W83, kindly provided by Dr. Margaret Duncan (The Forsyth Institute, Cambridge, MA, USA). The amplified gene was cloned into a pET21a plasmid between *NheI* and *XhoI* restriction sites. Domain fragments of *Pg*DPP III (1–679 aa) and AlkD alkylation domain like fragments (648–886, 660–886 and 675–886 aa) were cloned into a pLATE31 plasmid (aLICator kit, Thermo Fisher Scientific, Waltham, MA, USA). Replacement of Glu433 with Ala in the inactive variant (E433A) was performed according to instructions from the Q5^®^ Site-Directed Mutagenesis Kit (NEB). All constructs were cloned with a single C-terminal His tag. The primers, listed in S1 Table, were custom synthesized by Sigma Aldrich (St. Louis, MO, USA). Sequences of the cloned products were obtained with the automatic sequence analyzer ABI PRISM ^®^ 3100-Avant Genetic Analyser.

### Overexpression, purification, and characterization of recombinant DPP III proteins

Appropriate clones were transformed into the *Escherichia coli* expression strain BL21-CodonPlus(DE3)-RIL. The cells were grown in Luria Bertani broth with 100 μg/mL ampicillin. When the cells reached an OD_600_ of 0.6, the flasks were transferred to 16°C and induction of protein expression was carried out overnight by adding 0.1 mM IPTG (isopropyl β-D-1-thiogalactopyranoside). After harvesting, the cell pellets were resuspended in lysis buffer (20 mM NaPO_4_, 500 mM NaCl, 20 mM imidazole, 10 mM 2-mercaptoethanol, 1% glycerol, pH 8.0) and lysed by adding lysozyme followed by sonication on ice. A first purification step was done using Ni-NTA affinity chromatography according to Jajčanin-Jozić et al. [30], eluting the protein with 500 mM imidazole. Subsequent purification steps involved gel filtration on a HiLoad 16/60 Superdex 200 Prep grade column, followed by anion exchange on a HiPrep Q HP 16/10 column. Protein purity was confirmed by SDS-PAGE.

Secondary structure and temperature stability of recombinant proteins were determined by recording circular dichroism spectra (CD) on a Jasco J-815 spectropolarimetar (JASCO, Easton, MD, USA) with automatic temperature control according to Jajčanin-Jozić et al. [30]. Analysis of protein secondary structure from CD spectra was performed by using the CDSSTR program at the DICHROWEB site (http://public-1.cryst.bbk.ac.uk/cdweb/html). Protein concentrations were determined by the Bradford method [31].

### Enzyme activity determination and kinetic analysis

DPP III activity was determined by a standard colorimetric assay described previously [32] using Arg-Arg-2-naphthylamide (Arg_2_-2NA) and Arg-Arg-7-amido-4-methylcoumarin (Arg_2_-AMC) as substrates, at 37°C and pH 8.0, in 50 mM Tris-HCl buffer (I = 0.01) containing 100 μM CoCl_2_. For the determination of kinetic parameters (*K*_m_ and *k*_cat_) for the hydrolysis of the diarginyl substrates, initial hydrolysis rates were measured fluorimetrically at 25°C and at pH 8.0 in the presence of 100 μM CoCl_2_ [32]. The kinetic parameters were determined from the initial reaction rates, using a nonlinear regression program (GraphPad Prism version 5.04; GraphPad, La Jolla, CA, USA).

### Isothermal titration calorimetry (ITC)

Microcalorimetric data for ligands binding to the inactive E433A variant were measured in 50 mM Tris-HCl pH 8.0, 100 mM NaCl with a VP-ITC microcalorimeter (MicroCal, Northampton, MA, USA) equilibrated at 25°C. For this purpose, all ligands were dissolved in the same buffer as that of the purified enzyme. Both protein and ligand solutions were degassed immediately before the measurement. A titration experiment involved 30 injections using a 400 μM solution of peptide in the syringe against a 40 μM solution of *Pg*DPP III E433A in the measuring cell. In a standard experiment, a total of one aliquot of 2 μL and 29 aliquots of 10 μL of the peptide solution were injected into 2 mL of the protein solution under constant stirring at 270 rpm. Every injection was carried out over a period of 20 s with a spacing of 225 s between the injections. The corresponding heat of binding was calculated by integrating the area under each observed peak in the thermogram. A reference measurement where the peptide was injected into the buffer was performed as mentioned above and was subtracted to correct for the heat of dilution of the peptide. Nonlinear least-squares fitting using Origin version 7.0 (MicroCal) was used to obtain association constants (*K*_a_), heats of binding (ΔH) and stoichiometries. All measurements were made in duplicate.

### Small angle X-ray scattering (SAXS)

Data collection for the SAXS studies was performed using a PILATUS 1M Image Plate detector at the BM29 BIOSAXS beamline at the European Synchrotron Radiation facility (ESRF), Grenoble, France. The distance between the sample and the detector was 2.5 m. The protein samples for full-length *Pg*DPP III were measured at three different concentrations: 8.8, 4.43, and 1.02 mg/mL, in 50 mM Tris-HCl, pH 8.0, 100 mM NaCl buffer. Bovine serum albumin (BSA) at a concentration of 4.5 mg/mL was used as standard solution. The program PRIMUS was used to perform data analysis; scattering from the buffer was subtracted as background from the protein measurements [33]. Data from multiple concentrations were merged for data analysis and the evaluation of the radius of gyration (Rg) and the forward scattering intensity (I(0)) was performed using the Guinier approximation [34]. The pair distribution function was calculated with GNOM [35]. The theoretical scattering curve based on the atomic structure of the protein was calculated using CRYSOL and the subsequent calculation of the pair distribution function was performed using GNOM [35–37].

### HPLC-MS analysis

HPLC—MS analyses were carried out on an Agilent 1260 Infinity HPLC system (consisting of a G1311B quaternary pump, a G1329B autosampler, a G1316A thermostated column compartment and a G1314F variable wavelength detector) equipped with an Agilent ZORBAX SB-C8 column (15 mm × 4.6 mm, particle size: 3.5 μm) and coupled to an Agilent 6120 quadrupole LC—MS detector. The column effluent was split using a standard T-piece to allow the simultaneous recording of UV/Vis and MS signals. Method ‘GRADIENT_A-B_PEPTIDE_POS’: Eluents: water cont. 0.1% formic acid (A), acetonitrile cont. 0.1% formic acid (B); gradient elution program: 10% to 60% B over 25 min, 60% B for 1 min, 60% to 10% B over 4 min, 10% B for 5 min; injection volume: 5 μL; UV/Vis detection wavelength: 215 nm; MS ionization mode: ESI, pos.; MS spray chamber settings: drying gas temperature: 300°C, drying gas flow 10 mL/min, nebulizer pressure: 35 psig, capillary voltage 3000 V; MS signal 1: scan m/z = 100–1100, step size: 0.1, fragmentor voltage: 150 V, cycle time: 80%; MS signal 2: SIM on sample target masses, fragmentor voltage 150 V, cycle time: 20%. The HPLC-MS analysis was performed to determine if the peptides act as substrates or inhibitors. For this purpose, a mixture of 200 μL containing 1 mM solution of the corresponding peptide and 0.15 mM solution of *Pg*DPP III was incubated for 24 hours at room temperature and then analyzed using the method described above. Additionally, time series measurements were performed to obtain the velocity of angiotensin II degradation. For this purpose, a mixture of angiotensin II (1 mM) and *Pg*DPP III (0.15 mM) was incubated and the reaction was stopped using HPLC-graded acetonitrile at 5′, 30′, 1h, 2h, 3h, 4h, 5h, 6h and 24h. Corresponding time series were recorded for the degradation of angiotensin II by human DPP III which was expressed and purified as described previously [38].

### MMS complementation assay

The *B*. *cereus* plasmid pUC18alkD and the DNA glycosylase-deficient strain *E*. *coli* BK2118 (*tag*, *alkA*) were a kind gift from Magnar Bjørås (Oslo University Hospital/University of Oslo). Full-length *P*. *gingivalis* DPP III, the C-terminal AlkD like domain (648–886 aa), and *P*. *gingivalis* AlkD (Uniprot code Q7MV52) were subcloned into the pUC18 plasmid between *EcoR*I and *Pst*I restriction sites. Primers are listed in S1 Table. For plasmid propagation, *E*. *coli* TOP10 cells (Invitrogen, USA) were used. The complementation test was performed as described previously [39]. Briefly, *E*. *coli* BK2118 was transformed using Roti^®^-Transform (Roth) according to instructions, using the pUC18 constructs. We used an empty pET21a plasmid as a negative control, and the original *B*. *cereus* pUC18*alkD* as a positive control. BK2118 clones complementing the alkylation-sensitive phenotype were selected on Luria—Bertani (LB) agar plates containing 1, 2 or 5 mM of the alkylating agent methyl methanesulphonate (MMS) and ampicillin at a concentration of 50 μg/mL. From selected clones, overnight cultures were prepared and the next day serial dilutions of bacteria were plated on LBA/MMS plates. These plates were incubated for 2 days at 37°C.

### Computational methods

#### Bioinformatics and homology modelling

As the 3D structure of the DPP III from *P*. *gingivalis* has not yet been determined experimentally, we resorted to comparative modelling. The sequence was retrieved from the UniProt database (http://www.uniprot.org) and the domain structure and organization of *Pg*DPP III was predicted using two different approaches, the web server Phyre2, http://www.sbg.bio.ic.ac.uk/~phyre2 [40] and the stand-alone program Modeller9 [41,42]. The model was built using two templates. The model of the 3D structure of the DPP III domain (amino acids 1–659) was determined using the experimentally determined structure of *Bt*DPP III (PDB_code: 5NA7) as a template using the Phyre2 server. The sequence similarity between *Bt*DPP III and the DPP III domain of *Pg*DPP III is 51%. In order to identify a suitable template for the ARM domain a PSI-BLAST search was done using the C-terminal region (amino acids 660–886). A multiple sequence alignment was done using Clustal O 1.2.1 [43] at http://www.ebi.ac.uk/Tools/msa/clustalo/. Ultimately, the model of the ARM domain (660–886 aa residues) was determined using the program Modeller with the experimentally determined structure of AlkF from *B*. *cereus* (PDB_code 3ZBO, sequence similarity 15%) as a template.

#### Molecular dynamics, system parametrization and preparation

The zinc ion was added into the structure of *Pg*DPP III determined by homology modeling according to its position in *Bt*DPP III. Since the mode of Zn^2+^ binding is highly preserved in all DPP III orthologues we consider such a procedure justified. The obtained structure was used as the initial structure for MD simulations. The protein parametrization was performed within the ff14SB [44] force field using leap, a basic preparation program for Amber simulations available within the AMBER16 package (http://ambermd.org) [45]. For the zinc ion, parameters derived in previous work were used [46]. All Arg and Lys residues in the structure were positively charged (+1e) while Glu and Asp residues were negatively charged (-1e), as expected at physiological (experimental) conditions. The protonation of histidines was checked according to their ability to form hydrogen bonds with neighbouring amino acid residues or to coordinate the metal ion. The substrates were parameterized within the generalized amber force field (gaff) [47] and the missing parameters were derived using the Antechamber module [48] from the Amber16 suite of programs.

The proteins and protein-substrate complexes, were placed in a truncated octahedron box filled with TIP3P water molecules [49], and Na^+^ ions [50] were added in order to neutralize the systems.

#### MD simulations

Before running productive molecular dynamics simulations, the protein geometry was optimized in three cycles (each 1500 steps) and the system was equilibrated. In the first cycle of optimization, water molecules were relaxed, while the rest of the system was harmonically restrained with a force constant of 32 kcal mol^-1^ Å^-1^. In the second and third cycle, the same force constant (32 kcal mol^-1^ Å^-1^) was applied to the zinc cation, while the protein backbone was restrained with force constants of 12 and 2 kcal mol^-1^ Å^-1^, respectively. The energy minimization procedure, consisting of 470 steps of steepest descent followed by conjugate gradient optimization for the remaining steps, was the same in all cycles. During the first period of equilibration (200 ps of gentle heating from 0 to 300K with a time step of 1 fs), the *NVT* ensemble was used, while all of the following simulations were performed at constant temperature and pressure (300K and 1 atm, the *NpT* ensemble). During equilibration, the zinc ion and/or its ligands were weakly restrained. The temperature was held constant using a Langevin thermostat [51] with a collision frequency of 1 ps^−1^. The pressure was regulated by a Berendsen barostat [52]. Bonds involving hydrogen atoms were constrained using the SHAKE algorithm [53]. The ligand free protein was equilibrated for 50 ns (with time steps of 1 fs and 2 fs, for the first 1.5 ns and the remaining 48.5 ns, respectively), while the sampling was performed during the 150 ns, productive MD simulation. Furthermore, the structure of the ligand free protein, obtained by ligand extraction from the *Pg*DPP III-Arg_2_-2NA complex after a 100 ns simulation, was used for two additional, 150 ns MD simulations of the ligand free protein.

#### Docking

The structure obtained after 200 ns (50 ns of equilibration + 150 ns of productive MD) of MD simulation of the initial homology model of *Pg*DPP III was used to build the *Pg*DPP III substrate complexes. The *Pg*DPP III—Arg_2_-2NA and *Pg*DPP III—Arg_2_-AMC complexes were constructed using utilities of the program Pymol (The PyMOL Molecular Graphics System, Version 1.7 Schrödinger, LLC). The crystal structure of the tynorphin complex of the E451A variant of human DPP III (PDB code: 3T6B) was used as a template. Arg_2_-2NA was aligned to the bound tynorphin and then manually adjusted to avoid clashes with the enzyme. Obtained complex was energy minimized and equilibrated using the same procedure as described above for the ligand free enzyme. Since His437 moved away from the zinc ion during the equilibration, we used steered MD simulations (with a pulling force of 50 kcal mol^-1^ A^-1^) to bring it back. The thus obtained structure was again equilibrated for 30 ns (time step 1 fs) and two replicas of the *Pg*DPP III—Arg_2_-2NA complex were simulated at constant temperature and pressure (300K and 1 atm, the *NpT* ensemble, time step 2 fs) one for 200 ns and the other for 150 ns. The *Pg*DPP III—Arg_2_-AMC complex was built from the equilibrated structure of the *Pg*DPP III—Arg_2_-2NA complex. After energy minimization and a short equilibration (40 ps) its trajectory was simulated for 150 ns.

#### Data analysis

In order to analyze and characterize the conformational space that *Pg*DPP III structures span, as well as to determine the most relevant motions associated with protein closure, several types of data analysis were performed. All calculations were performed with the CPPTRAJ module of the Amber14 program package [45].

### Hydrogen/deuterium exchange (HDX) analysis

Hydrogen/deuterium exchange experiments (HDX) were performed as described previously [54]. A stock solution of 45 μM *Pg*DPP III in 20 mM Tris HCl, pH = 7.4 was prepared as well as the exchange buffer of the same composition and pH in D_2_O. H/D exchange reactions were carried out at room temperature and were started each time by diluting 5 μL of the stock solution into 45 μL of the exchange buffer. Reactions were performed in triplicate for incubation periods of 10 sec, 1 min, 20 min, 1 h and 4 h, each followed by acid quenching (adding 10 μL of 2 M glycine, pH = 2.5) and on-line pepsin proteolysis for 1.5 min. Deuterium uptake was measured for 96 non-overlapping peptic peptides covering 89% of the *Pg*DPP III amino acid sequence. The deuterium content (D) of those peptides was calculated by taking into account gains and losses of deuterons during digestion and the HPLC-MS measurement. An adjustment was made as proposed by Z. Zhang et al. [55] using control experimental data for non-deuterated and fully deuterated samples.

#### Correlation between HDX and MD simulation data

Comparisons of the experimental values for the deuterium content of peptides at each incubation time with corresponding values predicted by MD simulations were carried out similar to that described previously [56]. Briefly, 50 ns long fragments of the MD-trajectories were sampled at every 1 ps resulting in 50000 snapshots of the *Pg*DPP III structure. Simulations starting directly from the homology model and the simulations of the *Pg*DPP III extracted from the 150 ns simulated *Pg*DPP III—Arg_2_-2NA complex were used for the comparison.

Open state of the amide hydrogen for hydrogen/deuterium exchange reaction is defined as the number of snapshots where either NH or CO comes into contact with a water molecule. Closed state is defined as the number of all other snapshots. An ‘in house’ program was written in C# to detect backbone amide hydrogen bonding statistics by analysing frames of the MD trajectory. For each amide site the closed/open state ratio is calculated as follows: Closed/Open = (2*N(total)-N(NH-wat)-N(CO-wat))/(N(NH-wat)+N(CO-wat)). N denotes number of the snapshots with the characteristics specified in the brackets, for example N(NH-wat) is the number of frames in which the amide nitrogen is hydrogen bonded to at least one water molecule. The calculated ratio is directly used as the amide site protection factor (PF) without any specific mapping function. Intrinsic chemical rates (*k*_int_) for the hydrogen/deuterium exchange reaction of backbone amides are determined according to the procedure of Bai et al. [57]. In the case of *Pg*DPP III, intrinsic rate constants were obtained as for poly-DL-alanine in D_2_O at 20°C and pD_corr_ = 7.4 and non-blocked terminal amino acids. The calculation was done using the program Sphere (http://landing.foxchase.org/research/labs/roder/sphere) with default values for pK_a_ and activation energy. Rate constants for each amide hydrogen site were obtained as: *k*_pred_ = *k*_int_/*PF*. The deuterium content for each *Pg*DPP III peptide was calculated as follows:

$$D_{pep}=\sum_{j=m+1}^{n}\left(1-e^{\left[{-ki}_{j}/{PF}_{j}\right]}\right)$$

Summing amide hydrogen sites contributions within a peptide starts from the first residue next to the N-terminus and ends with the C-terminal residue.

## Results

### *Porphyromonas gingivalis* peptidase M49 is an atypical DPP III with a C-terminal extension exhibiting an ARM-type fold

All characterized DPPs III (peptidases of the M49 family) are composed of 675–786 amino acids [20,22]. However, peptidase M49 from *P*. *gingivalis* W83 (*Pg*DPP III) is an 886 amino acid residues long protein, 211 amino acids longer at the C-terminus than DPP III from *B*. *thetaiotaomicron*, and 149 amino acids longer than human DPP III.

We submitted sequences of the full-length protein (*Pg*DPP III), the DPP III fragment (amino acids 1–659) and the C-terminal fragment (amino acids 660–886) to the prediction servers HHPred and Phyre2 [58,40]. Interestingly, whereas for the N-terminal DPP III fragment a typical M49 family fold was predicted as expected, the C-terminal fragment was predicted at high confidence to have an alpha-alpha superhelix fold, belonging to the Armadillo (ARM) type fold family of alkylpurine DNA glycosylase AlkD (Phyre2: confidence = 100%) (S2 Fig). HHPred also predicted two domains in *Pg*DPP III: an N-terminal DPP III domain and a C-terminal AlkF/D domain (100% probability, E-value 2.1–7.1 E-31 for DNA glycosylase domain).

Genes coding for a fusion protein of dipeptidyl-peptidase III and an AlkD-like domain were found only within the *Porphyromonas* genus (Conserved Domain Database, February 2017, [59]). In *Bacteroides* and *Prevotella* genomes, on the other hand, we found these domains in close proximity, in the same order as in *Porphyromonas*, but as two separate genes (SyntTax, February 2017, http://archaea.u-psud.fr/synttax/).

### Physicochemical and catalytic properties of the purified recombinant proteins

We first expressed and purified full-length *Pg*DPP III protein. As we found structural similarity of the C-terminal fragment of *Pg*DPP III with members of the DNA glycosylase family, we also produced the DPP III and C-terminal domains in isolation, for subsequent investigation.

**Fig 1:**
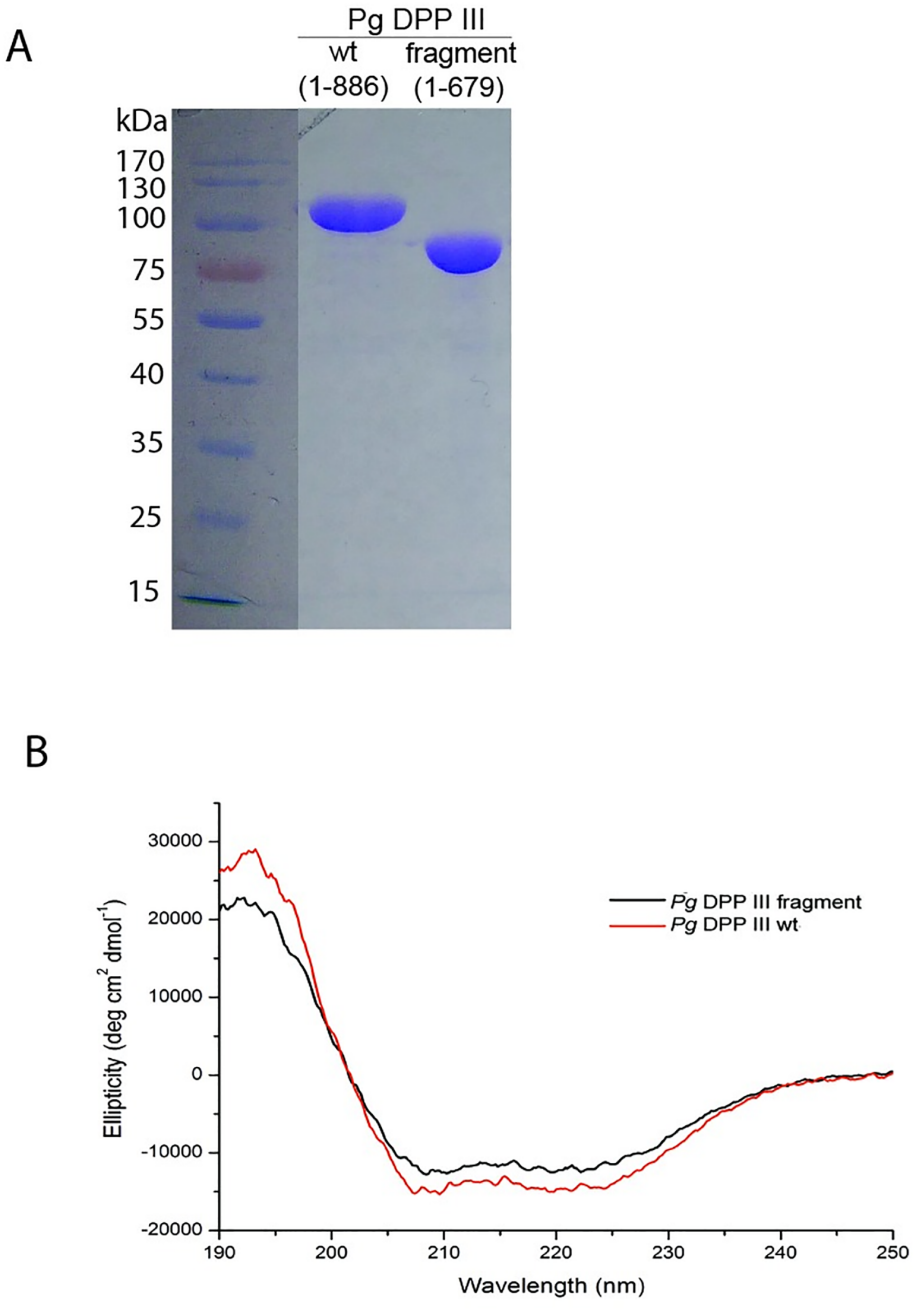
A) SDS page analysis of purified recombinant *Pg*DPP III wt (1–886 aa, left and the N-terminal DPP III fragment (1–679 aa, right; B) Far-UV spectra of the full length protein and the DPP III fragment.

Cloning, expression, and purification of the recombinant proteins was performed as described in the Materials and methods section. Enzyme activity was assayed using a set of synthetic dipeptide-2-naphthylamide substrates as described previously [60]. Full-length *Pg*DPP III and the DPP III fragment (a. acids 1–679) were purified to apparent homogeneity according to SDS-PAGE, with estimated molecular weights of 102000 and 78000, respectively, which was in agreement with their predicted molecular masses (Fig 1A). We cloned and attempted to express three C-terminal fragments of different sizes. Only one (amino acids 648–886) was detected on SDS-PAGE. However, it could not be purified as all expressed protein was in insoluble inclusion bodies. This problem was not solved by attempts to yield soluble protein by refolding from inclusion bodies.

Peptidase activity assays with different dipeptidyl-2NAs showed a similar substrate specificity of *Pg*DPP III as compared to *Bt*DPP III and several other members of the M49 family, with a preference for Arg_2_-2NA [22]. In addition, Phe-Arg-2NA and Ala-Arg-2NA were hydrolysed with high rates (S2 Table).

Deconvolution of the CD spectra using the CDSSTR program predicted 45.6% helices, 10.8% strands, 17.0% turns and 26.8% of unordered secondary structure for full-length *Pg*DPP III (Fig 1B). Comparing the CD spectra of full-length *Pg*DPP III and of the DPP III fragment, as well as thermal denaturation curves (showing a T_m_ of 50°C in both cases) revealed no significant difference in secondary structure or temperature stability. However, the specific activity of the DPP III fragment for the hydrolysis of Arg_2_-2NA was 250-fold lower compared to the full-length protein. We also determined the kinetic parameters of both the full-length enzyme and the DPP III fragment for the hydrolysis of the fluorogenic substrates Arg_2_-2NA and Arg_2_-AMC. As shown in Table 1, this kinetic analysis revealed pronounced differences between full length *Pg*DPP III and the DPP III fragment. The *K*_m_ values for both substrates were increased by 4-fold when the C-terminal domain was removed. A striking difference was also observed in the *k*_cat_ values which were reduced 120-fold in the case of Arg_2_-AMC, and 250-fold in the case of Arg_2_-2NA, resulting in a 470-fold and 1050-fold reduction in the catalytic efficiency, respectively.

**Table 1:** Kinetic analysis of full length *Pg*DPP III and its DPP III fragment.

**Full length *Pg*DPP III** (1–886 aa)			
**Substrate**	***K***_**m**_**(μM)**	***k***_**cat**_ **(s**^**-1**^**)**	***k***_**cat**_**/*K***_**m**_ **(mM**^**-1**^ **s**^**-1**^**)**
Arg-Arg-2NA	0.97 ± 0.16	0.75 ± 0.13	773.20
Arg-Arg-AMC	2.56 ± 0.32	0.29 ± 0.11	113.28
**DPP III fragment** (1–679 aa)			
**Substrate**	***K***_**m**_**(μM)**	***k***_**cat**_ **(s**^**-1**^**)**	***k***_**cat**_**/*K***_**m**_ **(mM**^**-1**^ **s**^**-1**^**)**
Arg-Arg-2NA	4.37 ± 0.42	0.0032 ± 0.0005	0.732
Arg-Arg-AMC	9.97 ± 0.44	0.0024 ± 0.0014	0.241

Enzyme concentration was 0.5–1 nM for full length *Pg*DPP III and 40–100 nM for the DPP III fragment. The fluorescence of the liberated product 2-naphthylamine was measured at 26°C and pH 8.0 (Tris-HCl buffer ionic strength: 0.01M) in the presence of 100 μM CoCl_2_. Mean values of at least two independent experiments ± standard deviation are given.

### Interaction with peptides

To investigate interactions of the inactive E433A variant of full-length *Pg*DPP III with peptides, we chose three model peptides, which enable comparison to eukaryotic DPPs III: the pentapeptides tynorphin (VVYPW) and IVYPW, and the octapeptide angiotensin II (DRVYIHPF).

**Fig 2:**
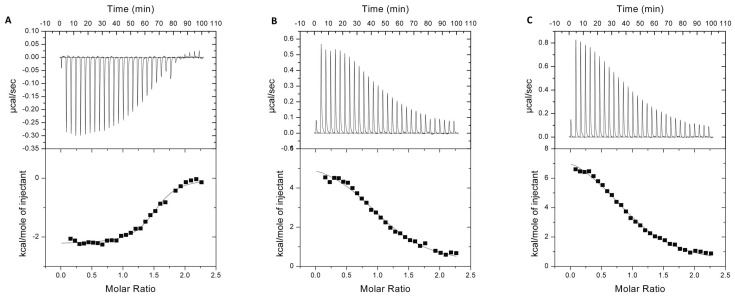
Isothermal titration calorimetry measurements of the E433A variant of *Pg*DPP III with A) angiotensin II, B) tynorphin and C) IVYPW. The upper panels show the time course evolution of heat for each injection and the bottom panels show peak integration as a function of molar ratio of E433A *Pg*DPP III (inactive variant) to all three ligands. The data were corrected by subtraction of an appropriate blank experiment and fit with nonlinear regression. The solid curves represent the best fits using a one binding site model.

We performed isothermal titration calorimetry in order to determine the binding affinity. As in previous studies [26,38], we chose the inactive variant for our experiments, in order to avoid contamination of the calorimetric data by heat or reactions resulting from peptide hydrolysis. In the case of tynorphin and IVYPW, we obtained the same endothermic mode of binding as previously described for human DPP III [26], while the binding of angiotensin II was exothermic (Fig 2). The thermodynamic parameters obtained from the ITC experiments are presented in S3 Table. It was shown that angiotensin II has stronger affinity compared to the other two peptides. Binding thermodynamics of these peptides to human DPP III have previously been investigated [26,38]. Overall, the human enzyme exhibits tighter binding to all three peptides, when compared to *Pg*DPP III.

### Hydrolytic activity towards peptides

In a first set of biotransformation experiments wild type *Pg*DPP III (0.16 mM) was incubated for 24 h separately with the three oligopeptides (1 mM) that showed binding to the protein in ITC experiments: angiotensin II, tynorphin and IVYPW. HPLC—MS analyses confirmed that all three peptides are substrates of *Pg*DPP III (S3 Fig). In all reaction mixtures, the original peptide was completely consumed, while no conversion could be detected in blank experiments in the absence of *Pg*DPP III. The main reaction product in the case of angiotensin II was the C-terminal tetrapeptide IHPF (retention time, *t*_R_ = 6.0 min; m/z [M+H] ^+^ = 513.3). In addition, a product with a very similar mass spectrum but a different retention time (*t*_R_ = 5.5 min) was formed in minor amounts. This side product is likely the *cis*-prolyl isomer of IHPF, which is in agreement with the observation that also the HPLC—MS chromatogram of angiotensin II itself shows two peaks of the same mass (*t*_R_ = 7.9 min, 8.2 min), indicating that the substrate is also present as a mixture of prolyl isomers.

The cleavage of the other two substrates, tynorphin and IVYPW, by *Pg*DPP III led to the C-terminal tripeptide YPW (*t*_R_ = 9.1 min, m/z [M+H]^+^ = 465.3) as the main product. Interestingly, the C-terminal dipeptide (PW; *t*_R_ = 6.1 min, m/z [M+H]^+^ = 302.2) was also formed as a minor side product in both cases. An additional compound observed in the biotransformation of IVYPW (*t*_R_ = 9.9 min, m/z [M+H+] = 475.3) was detectable only by MS but not by UV and therefore could not be identified (S3 Fig).

In view of the known manner of enzymatic action of dipeptidyl peptidases, the formation of a C-terminal tetrapeptide from the octapeptide angiotensin II is most likely the result of two consecutive N-terminal dipeptide cleavages. To substantiate this assumption, we carried out a biotransformation of angiotensin II with a significantly lower concentration of *Pg*DPP III (2 mg/mL; 0.018 mM), and we followed the reaction time course to identify potential reaction intermediates. Two interesting observations were made in this experiment (S4 Fig). Firstly, the major isomer of angiotensin II (*t*_R_ = 8.1 min) is converted much faster than the minor one (*t*_R_ = 8.3 min). As a consequence, the isomeric ratio drops from 23:1 at the start of the reaction to 6:1 after 1 h. After 2 h the main isomer is no longer detectable and after 4 h the minor isomer is completely consumed. Secondly, the hexapeptide VYIHPF, which is, besides the N-terminal dipeptide (DR), the product of the first enzymatic cleavage and can hence be considered a reaction intermediate, could indeed be detected (*t*_R_ = 9.1 min, m/z [M+H] ^+^ = 775.4). Its concentration increased during the first 30 min of the reaction and decreased afterwards.

For comparison, angiotensin II was incubated with human DPP III and this reaction mixture was analyzed by HPLC-MS. As shown in S4 Fig, the human enzyme hydrolyzed the peptide in the same way as *Pg*DPP III, only faster. This confirms angiotensin II is a good substrate for human DPP III.

### Complementation assay in the DNA-repair-deficient *E*. *coli* strain

We predicted the C-terminal fragment of *Pg*DPP III has a similar fold to AlkD, a bacterial DNA glycosylase that removes positively charged methylpurines from DNA [61].

*E*. *coli* strain BK2118 is extremely sensitive to alkylating agent methyl methanesulphonate (MMS), because it lacks both AlkA and Tag 3-methyladenine (3mA) DNA glycosylases, which makes this strain alkylation repair-defective [39]. Functional complementation of the *tag alkA* double mutant of *E*. *coli* with a gene expressing 3mA DNA glycosylase activity was shown to restore alkylation resistance [39]. Therefore, in order to investigate the potential DNA alkylation repair function of *Pg*DPP III, we transformed *E*. *coli* strain BK2118 with pUC18 constructs harbouring the *Pg*DPP III full length gene, the *Pg*DPP III C-terminal domain, AlkD from *Bacillus cereus* and AlkD from *P*. *gingivalis*. Transformants were plated in different concentrations and grown on media containing different concentrations of MMS (1 mM to 5 mM) for two days. Full rescue was obtained with plasmids expressing AlkD from *B*. *cereus* or AlkD from *P*. *gingivalis*, but not with full length *Pg*DPP III, indicating that *Pg*DPP III does not possess alkylation repair function (S5 Fig).

### Study of the structure and dynamics of the full-length *Pg*DPP III

To obtain deeper insight into the structural and dynamical properties of *Pg*DPP III as well as to elucidate its interactions with substrates (ligands), we used molecular modelling combined with hydrogen/deuterium exchange measurements.

#### Molecular modeling

Comparative modelling was used to derive a model of the *Pg*DPP III as described in the Computational methods section. The structure of the N-terminal DPP III domain was modelled using the crystal structure of DPP III from *B*. *thetaiotaomicron* as template, whereas the model of the C-terminal region (ARM domain) was derived using the structure of AlkF from *B*. *cereus* (PDB-code 3ZBO) as template.

This homology model (S6 Fig) enabled further computational studies of the structure and dynamics of ligand-free *Pg*DPP III as well as its complexes with the synthetic substrates Arg_2_-2NA and Arg_2_–AMC.

#### MD simulations of the ligand-free protein

**Fig 3:**
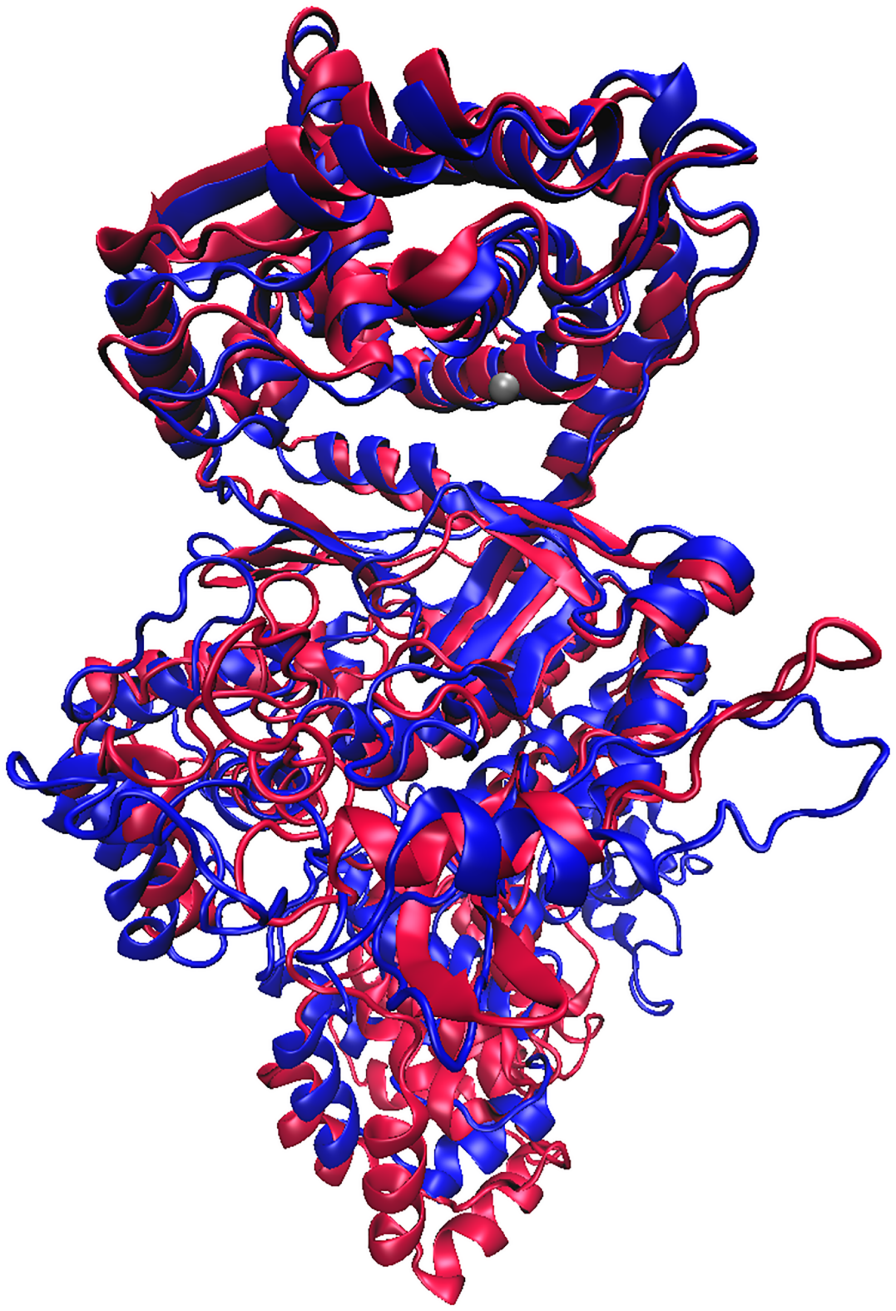
Overlay of the ligand free structures of *Pg*DPP III generated during MD simulations. Overlay of the initial structure of PgDPP III (obtained by comparative modelling, redand the structure obtained after 200 ns of MD simulations (blue). The zinc ion is represented as a grey sphere.

**Fig 4:**
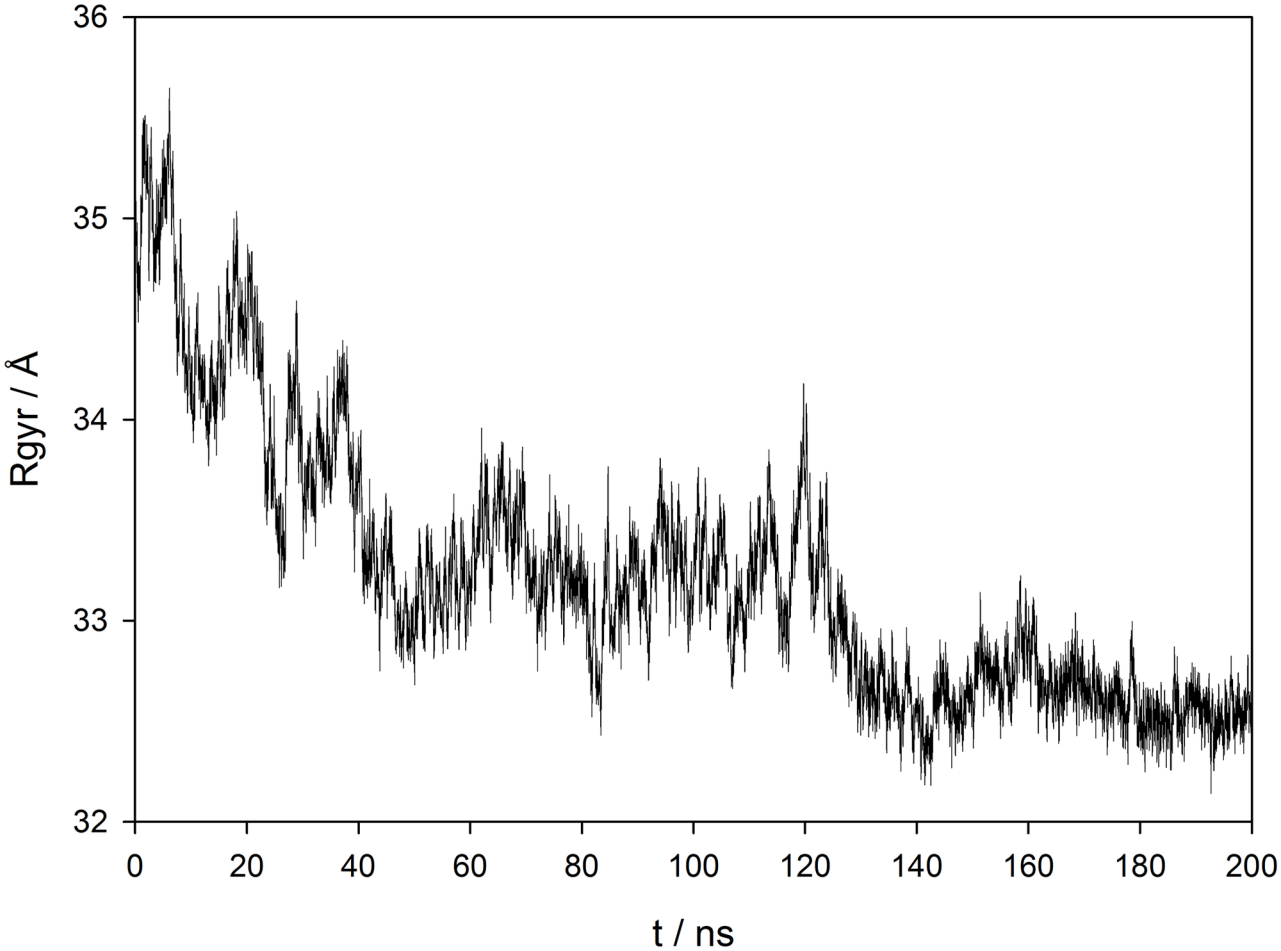
Radius of gyration profile. Radius of gyration (Å) profiles for the *Pg*DPP III structure during 200 ns of MD simulations (50 ns of equilibration + 150 ns of productive MD).

After energy-optimization and equilibration (50 ns) the structure was subjected to 150 ns of MD simulation at room temperature (details are given in the Methods section). During the MD simulations, the C-terminal, ARM region became more structured (share of helical structure increased from 30 to about 60%; Fig 3 and S4 Table) and the entire enzyme structure became more compact with the highest compression occurring during the first 50 ns (equilibration time; Fig 4). The separation between the two lobes of the DPP III domain decreased, as reported for the human orthologue [62], but also a reorientation of the ARM fragment relative to the DPP III domain occurred (Fig 3). However, the secondary structure within the DPP III domain, as well as the zinc ion coordination, was mostly preserved during the simulations.

**Fig 5:**
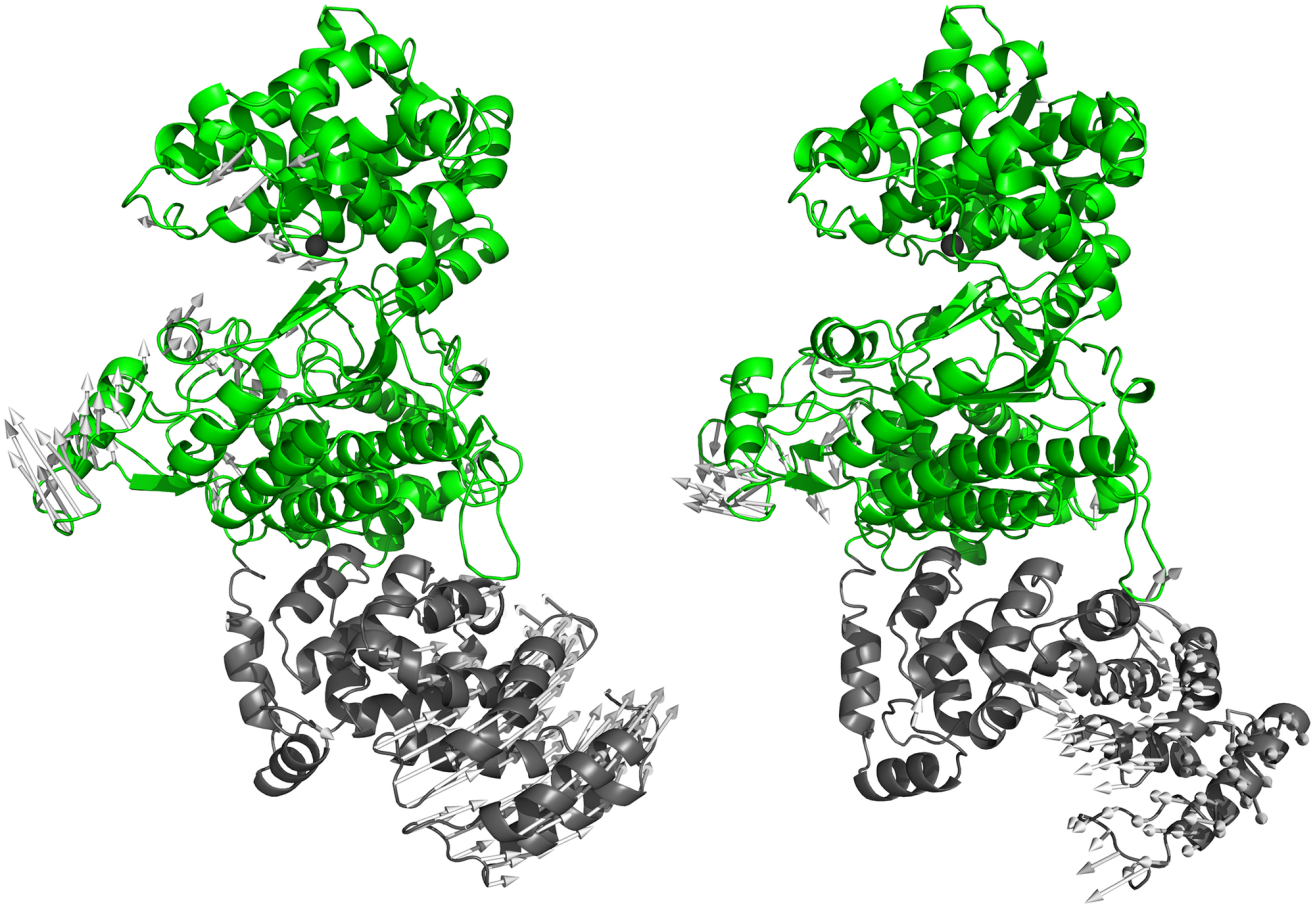
Motions described by the eigenvectors of the first (left) and the second (right) principal components. The components are derived from a principal component analysis of the 150 ns trajectory of ligand free *Pg*DPP III. The gray arrows attached to the each Cα atom indicate the eigenvector direction and magnitude of its value. The DPP III fragment is in colored green and the ARM fragment in gray.

A principal components (PC) analysis revealed two dominant components which explained ~94% of the total variance generated during MD simulations (PC1 describes 60.6%, and PC2 33.8% of the total variance). The most prominent motion, described by their eigenvectors corresponds to the closure of the DPP III fragment and to the overall protein compression. The first eigenvector describes displacement of the outer edges of the DPP III fragment cleft accompanied with rotation of the C-terminal ARM region in the direction of the N-terminal DPP III region (Fig 5). The second eigenvector describes a parallel shift of the lower DPP III domain and the lower part of the ARM fragment. The most prominent feature is the correlated motion of the lower DPP III domain and the ARM fragment.

**Fig 6:**
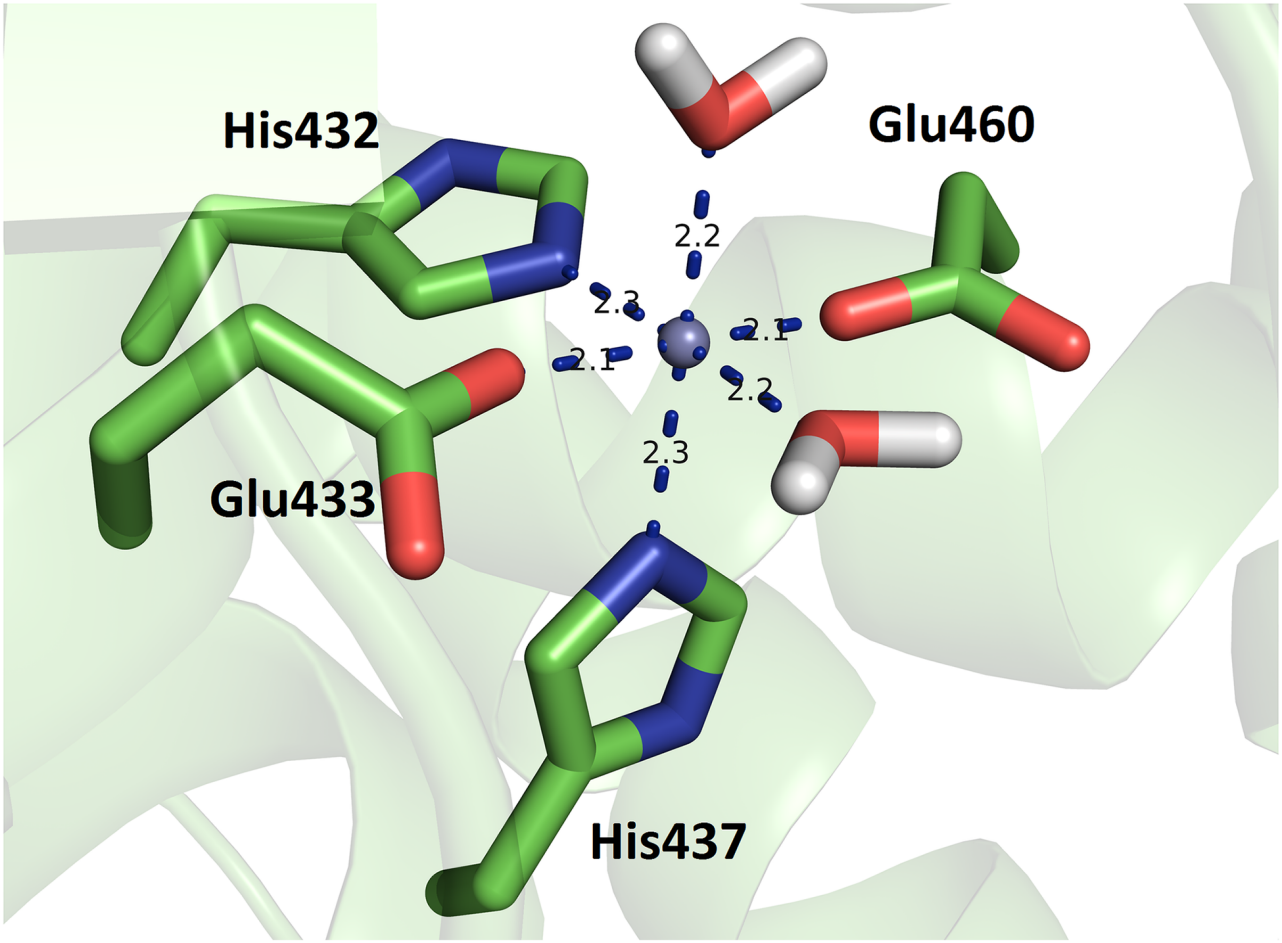
Typical mode of Zn^2+^ binding during simulations of the ligand free *Pg*DPP III. The coordination was accomplished with Nε atoms of the His432 and His437 imidazole rings, carboxylate oxygens of Glu460 and either Glu433 and two or three water molecules.

During the simulation, Zn^2+^ was mostly hexacoordinated. The coordination was accomplished with the Nε atoms of His432 and His437, the carboxylate oxygen atoms of Glu433 and Glu460, and two water molecules. The carboxylates of both Glu433 and Glu460 coordinated the metal ion monodentantely during the entire simulation. Occasionally, an additional water molecule replaced Glu433 in the zinc coordination sphere. A typical representation of metal ion coordination during the simulation is given in Fig 6. Our previous QM/MM study on human DPP III showed that a hexacoordinated zinc ion is energetically the most advantageous type of Zn^2+^ coordination in the open and semi-open conformations of hDPP III [63].

In addition to the homology model, the protein structure extracted from the 100 ns simulated *Pg*DPP III—Arg_2_-2NA complex (simulations of the complexes are given below) was used to study the behavior of ligand free *Pg*DPP III in water. During two 150 ns long MD simulations of this *Pg*DPP III structure neither the protein secondary structure (see S4 Table) nor the protein compactness changed significantly (Rgyr fluctuated between 31 and 32.5 Å). Also, the zinc ion coordination was similar to the coordination during previous, 200 ns long MD simulations of the homology derived *Pg*DPP III model (see S7 Fig).

#### MD simulations of *Pg*DPP III in complex with diarginyl arylamide substrates

The *Pg*DPPII binding site is situated deep in the interdomain cleft. It is mostly defined by the amino acid residues from the helices situated at the bottom of the DPP III upper domain, H11(410–436) and H12(452–473) and the beta strand, E7 (Ile366-Asn372/Asp374), from the upper part of the lower domain beta core.

The *Pg*DPP III—Arg_2_-2NA complex was built using the *Pg*DPP III structure obtained after 150 ns of the productive MD simulations of the ligand-free protein and the crystal structure of the tynorphin complex of the E451A variant of human DPP III (PDB code: 3T6B) as a template [26]. The subsequent obtained structure was equilibrated for 30 ns and two replicas of the *Pg*DPP III—Arg_2_-2NA complex were simulated, one for 150 ns and the other for 200 ns. During these simulations, the radius of gyration fluctuated between 31.5 and 32.5 Å (S8 Fig).

**Fig 7:**
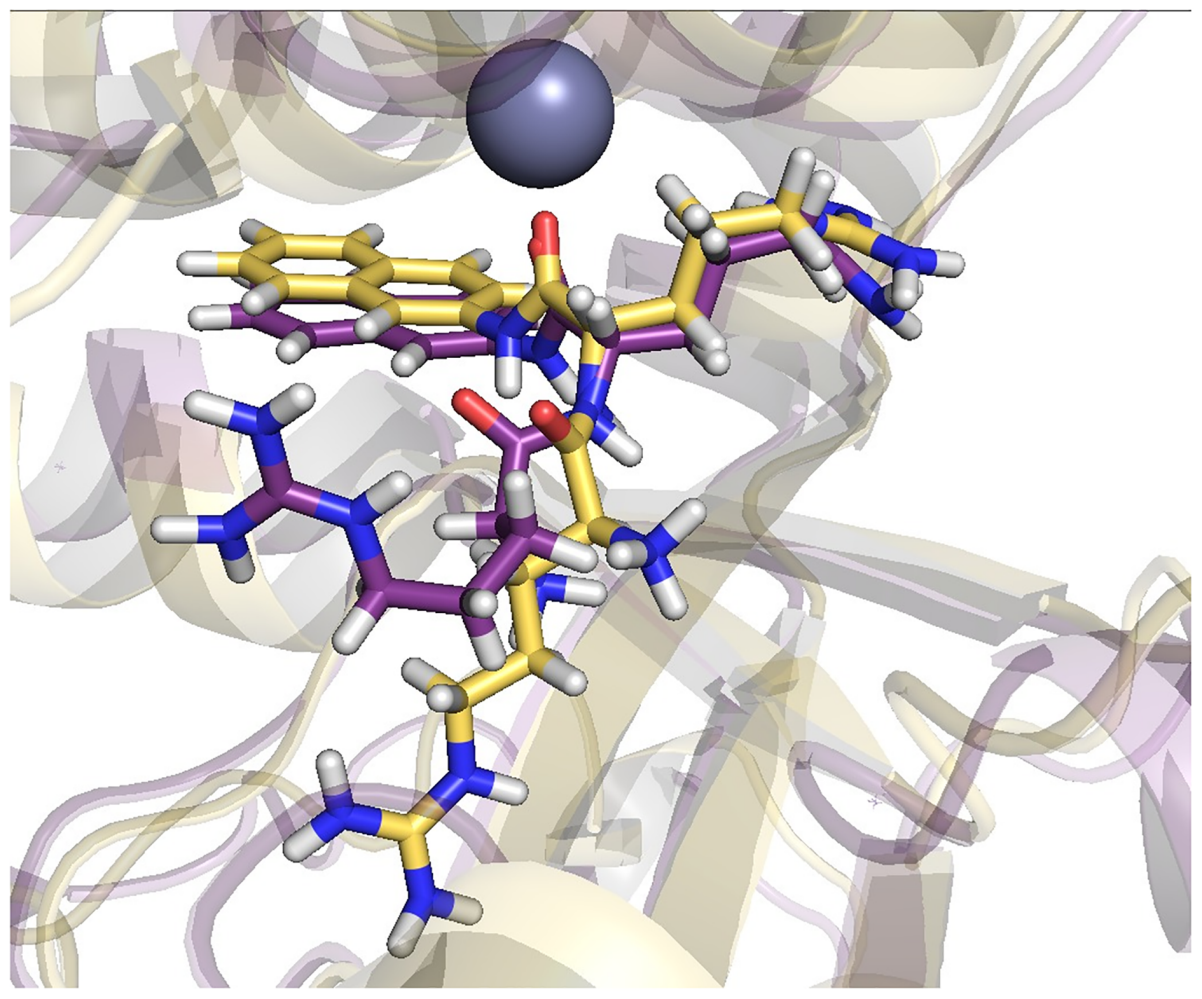
Orientation of Arg_2_-2NA in the active site of *Pg*DPP III determined by separate simulations of two replicas of the *Pg*DPP III-Arg_2_-2NA complex. The replica obtained at the end of 200 ns of MD simulation is colored yellow and replica obtained after 150 ns of MD simulations violet). The Zn^2+^ ion is represented as a gray sphere.

In the final structures, the substrate is stabilized with several strong hydrogen bonds and electrostatic interactions established with charged amino acid residues like Glu291, Glu304, Asp359, Asp374, Glu433 and Glu460 (S5 Table and S9 Fig). While the naphthylamide group mostly sat inertly in the large, partly hydrophobic pocket, where it interacts with Ala348, Ile366, Gly367 and Thr429, the arginine side chains changed their orientation during the MD simulations. Such behavior of the substrate is a consequence of the shape of the substrate binding pocket. Since the hydrophobic bottom end of the binding pocket is bottle shaped, rotations of the naphthylamide group are sterically hindered. On the other hand, the rest of the binding pocket is partially water exposed and relatively wide with the negatively charged regions dispersed around its surface (S10 Fig), which enables the positively charged arginine side chains to accommodate different orientations. It is therefore not surprising that in the final structures, the orientation of the side chain of the first Arg (from the N-terminus) is different in the two simulated replicas. An overlay of the active sites in the final structures obtained by MD is shown in Fig 7.

Zn^2+^ was coordinated by Nε atoms of the His432 and His437 imidazoles, the Glu433 and Glu460 carboxylate oxygens and by the carbonyl oxygen of the second arginine of the substrate during the simulations of both replicas.

**Fig 8:**
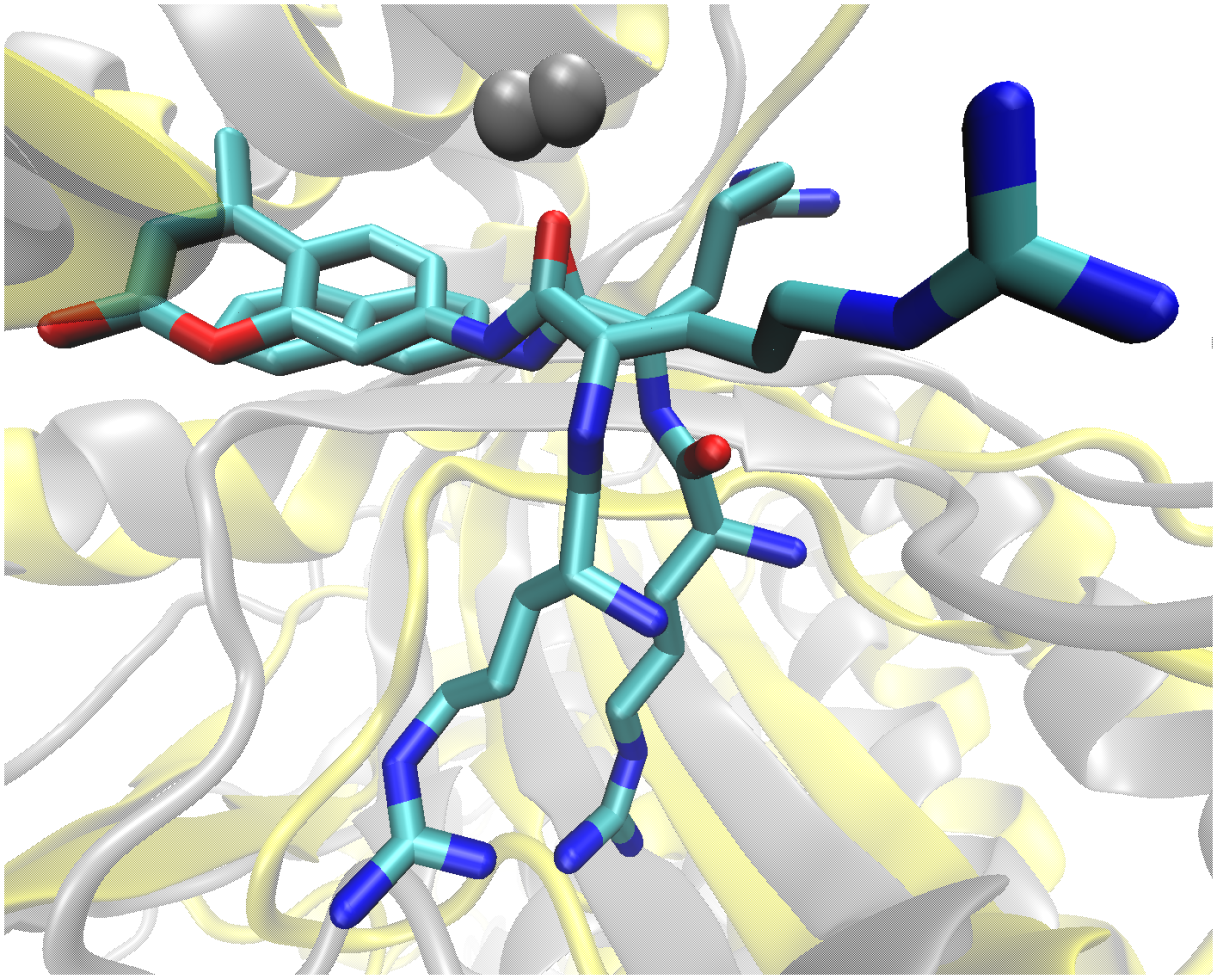
Overlay of Arg_2_-2NA and Arg_2_-AMC bound in the active site of *Pg*DPP III iThe final structures obtained by simulation of the *Pg*DPP III—Diarginyl arylamide substrates complexes are aligned. Substrates are shown as sticks representation and the protein structures as cartoon representations, colored yellow (*Pg*DPP III—Arg_2_-2NA, simulated 200 ns) and gray (*Pg*DPP III—Arg_2_-AMC, simulated 150 ns), respectively. The Zn^2+^ ion is represented as a gray sphere.

The *Pg*DPP III—Arg_2_-AMC complex was built from the equilibrated *Pg*DPP III—Arg_2_-2NA complex in a way that the naphthyl ring was replaced with AMC. It was simulated for 150 ns, during which the radius of gyration fluctuated between 31 and 32 Å.Similar to the *Pg*DPP III—Arg_2_-2NA complex, Zn^2+^ is coordinated by His432, Glu433, His437, Glu460 and the carbonyl oxygen of the second arginine of the substrate during the simulation (S11 Fig). In the final structure, the Arg_2_-AMC orientation in the enzyme active site is similar to that of Arg_2_-2NA in one (200 ns simulated) replica (Fig 8). However, the side chain of the second arginine from the N-terminus in Arg_2_-AMC is oriented differently than in Arg_2_-2NA and stabilized by an interaction with Asp374 (see S11 Fig for substrate binding and the Zn^2+^ coordination).

#### H/D exchange of the ligand-free protein

Earlier studies have shown that the hydrogen/deuterium exchange (HDX) approach can reveal data on protein structure and flexibility [56,64,65].

**Fig 9:**
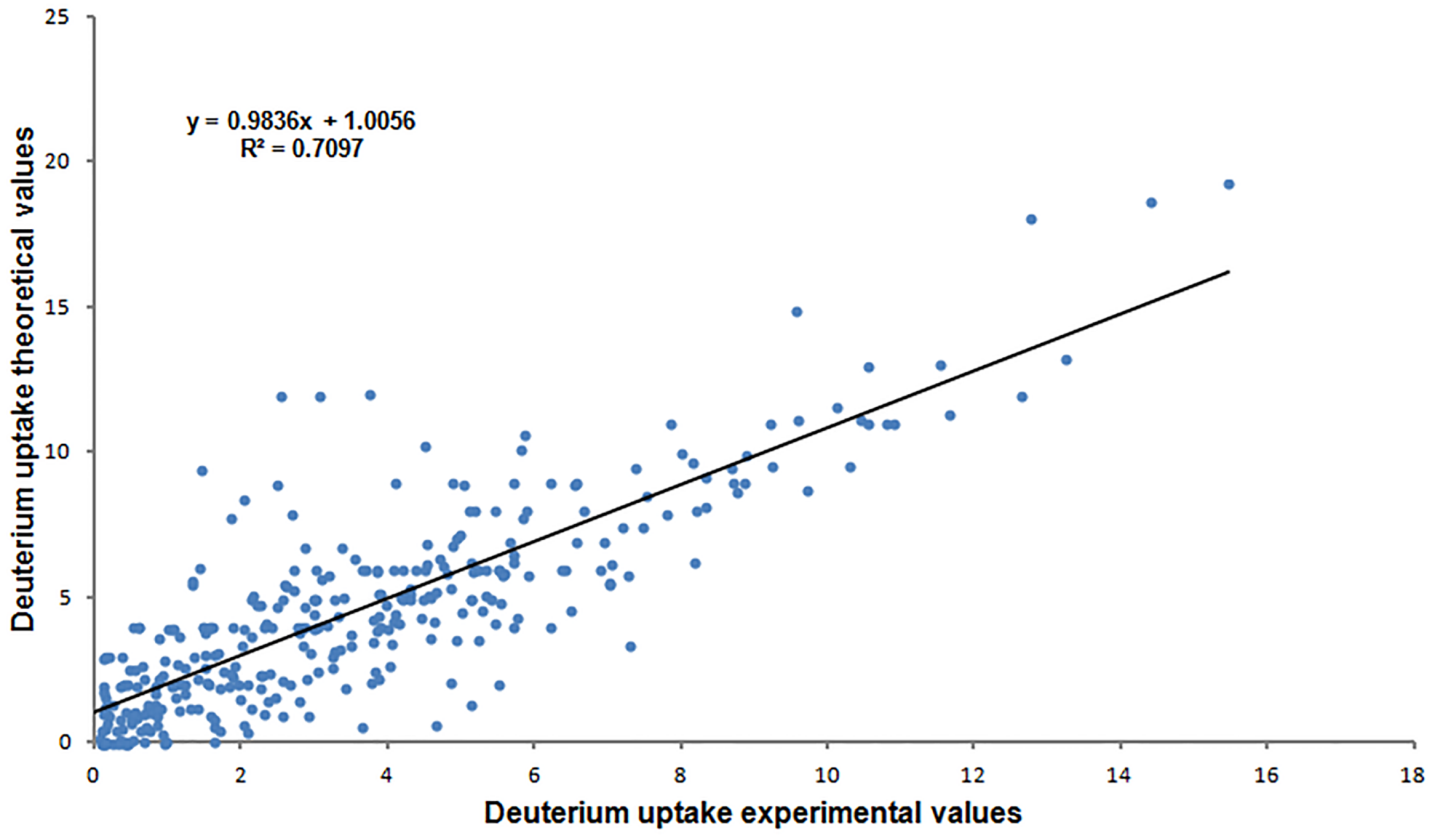
Correlation between HDX and MD simulation data. Correlation of the HDX results with the theoretical data based on the MD simulations of the *Pg*DPP III structure extracted from the simulated *Pg*DPP III—Arg_2_-2NA complex and simulated for 100 ns for the DPP III fragment of the *Pg*DPP III structure (amino acids 14–654).

In this work, we combined HDX results with data extracted from MD simulations in order to get detailed insight into protein structure and dynamics. The HDX results could be a good estimate of the MD simulations reliability. The results of the hydrogen/deuterium exchange experiment for every peptide at each incubation period were correlated with the results of MD simulations, where the open state of the amide hydrogen for hydrogen/deuterium exchange reaction is defined as the number of snapshots in which either NH or CO comes into contact with a water molecule and the ratio of an amide site closed/open states calculated by equation: Closed/Open = (N_total_-N_solvated_)/N_solvated_ was used as the amide site *j* protection factor (PF_*j*_) in the equation for the calculation of the peptide deuterium content *D*_*pep*_ (equation given in Material and methods). The overall correlation of the HDX data with the simulation results starting from the homology model of *Pg*DPP III is about 0.50, while the correlation with the longer simulated structure, namely the *Pg*DPP III structure extracted from the simulated *Pg*DPP III—Arg_2_-2NA complex is about 0.65 (S12 and S13 Figs). During the MD simulations in water, the *Pg*DPP III structure becomes more compact and, according to the results of comparison with the dynamical behaviour predicted by the HDX experiment, it is more reliable than the initial, extended structure determined by homology modelling. In both cases, a better correlation (about 0.53 and 0.71, S14 Fig and Fig 9, respectively) was obtained for the DPP III than for the ARM region.

#### SAXS

**Fig 10:**
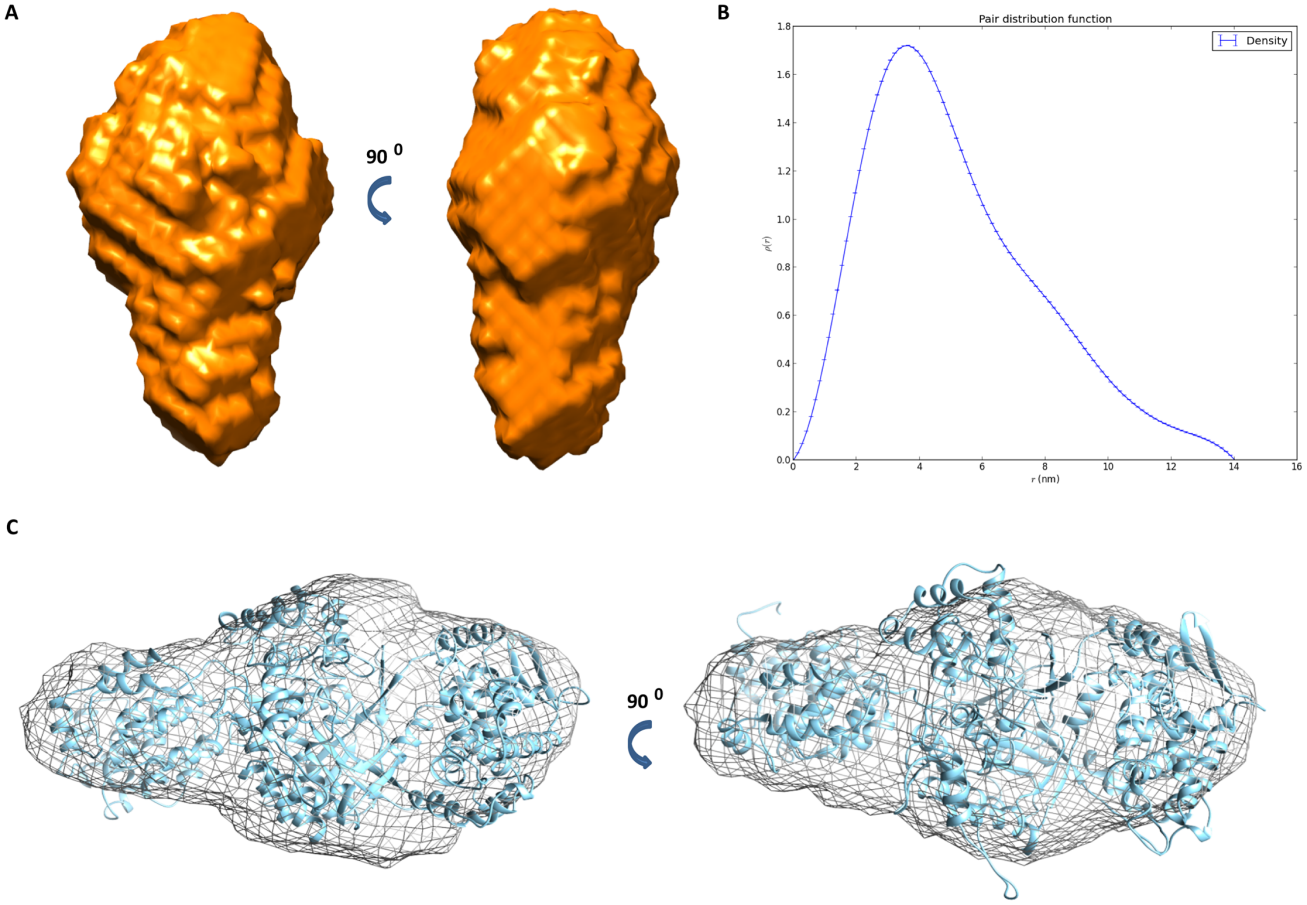
SAXS envelope of full-length *Pg*DPP III. (A) Surface representation of the low resolution SAXS structure. (B) The distance distribution function p(r) calculated using the program GNOM indicates an elongated, oval particle. (C) The homology model of full-length *Pg*DPP III (cartoon representation) modeled into the SAXS envelope (shown as a mesh object).

The homology model of full-length *Pg*DPP III was modeled into the SAXS envelope as a rigid body using the ‘fit in volume data‘-algorithm in Chimera (Fig 10). The molecular envelope of the protein was calculated from the scattering curves using the program DAMMIF, averaged with DAMAVER and refined with DAMMIN. According to the SAXS data, the protein is monomeric in solution and shows a molecular weight of 101 kDa (calculated using I(0) and compared to the I(0) value of BSA). The D_max_ was found to be 113 Å with a radius of gyration of 30 Å (deduced from a Guinier analysis). The distance distribution function p(r) calculated by GNOM points towards an elongated oval particle. The low resolution structure has been deposited into the SAXS database under the code SASDC58 [66].

## Discussion

Dipeptidyl peptidases of *P*. *gingivalis* have been the subject of extensive research because these exopeptidases liberate dipeptides from the amino-end of their natural substrates, and *P*. *gingivalis* is known to utilize dipeptides preferentially, instead of free amino acids, as the source of energy [28]. Until now, beside DPP IV, which is a proven virulence factor, three other DPPs were reported: DPP5, DPP7 and DPP11 [67–69]. They are all serine peptidases, localized in the periplasm, but differ in their substrate specificity [15]. In addition to DPPs, gingipains R and K were demonstrated to contribute to dipeptide production from extracellular oligopeptides [15]. Investigations with gene-disrupted mutants of *P*. *gingivalis* ATCC 33277 indicated that DPPs and gingipains cooperatively liberate dipeptides from nutrient oligopeptides [67]. In their search for an unidentified enzyme possessing a DPP7-like substrate specificity, Ohara-Nemoto et al. [67] also expressed the gene PGN_1645 coding for a putative DPP III from *P*. *gingivalis* strain ATCC 33277. They examined the substrate specificity of the recombinant protein with dipeptidyl-AMC substrates and found the highest activity for Arg-Arg-AMC. This protein is very similar to DPP III from *P*. *gingivalis* strain W83, described in our study, only 20 amino acids longer (906 a. acids), with 96% identity. The same authors have also shown that gingipain null-mutant KDP136 cells had defects in Arg-Arg-AMC hydrolysis, compared to the wild-type *P*. *gingivalis* ATCC 33277, and therefore suggested that DPP III did not participate in extracellular dipeptide production in *P*. *gingivalis*. This finding indicated that *P*. *gingivalis* DPP III is not a periplasmic enzyme. However, the probable cytosolic localization of *Pg*DPP III does not exclude its importance in protein metabolism (*i*.*e*. oligopeptide cleavage).

In this study we have characterized an atypical dipeptidyl peptidase III, the product of the gene PG_0317, using molecular biology, biochemical, biophysical, and computational chemistry methods. This 886 residues long protein contains an additional C-terminally appended domain predicted to possess the superhelical ARM-type fold.

Our investigation of the biochemical properties confirmed that *Pg*DPP III acts as a true dipeptidyl peptidase III, cleaving dipeptides sequentially from the N-terminus of an oligopeptide. *Pg*DPP III is capable of hydrolysing peptides of various compositions and length, like the octapeptide angiotensin II (DRVYHIPF) and its hexapeptide fragment VYIHPF, as well as the pentapeptides VVYPW (tynorphin) and IVYPD (Fig 2). Among the dipeptidyl-2NA substrates *Pg*DPP III prefers diarginyl-2NA (S2 Table).

Kinetic analyses of the hydrolysis of the preferred substrate Arg_2_-2NA showed similarities in *K*_m_ and *k*_cat_ values compared to *B*. *thetaiotaomicron* DPP III [24], and pronounced differences in comparison to the yeast enzyme [70]. The catalytic efficiency (*k*_cat_/*K*_m_) of full-length *Pg*DPP III is 50-fold higher than that of the yeast counterpart, due to the decrease in *K*_m_ (12-fold) and the increase (4.2-fold) in the *k*_cat_ value. In contrast, compared to human DPP III [70], the *k*_cat_/*K*_m_ value of the *P*. *gingivalis* enzyme is 12- and 26-fold lower for both Arg_2_-2NA and Arg_2_-AMC, respectively, predominantly owing to the difference in *k*_cat_ values.

Interestingly, Arg-Arg-2NA is a much better substrate for *Pg*DPP III than Arg-Arg-AMC (2.6-fold higher *k*_cat_, and 6.8-fold higher catalytic efficiency) (Table 1). Human DPP III also prefers Arg_2_-2NA over Arg_2_-AMC, but entirely on the account of a change in the *K*_m_ value [70].

Due to the high sequence similarity with *B*. *thetaiotaomicron* DPP III, we were able to obtain a homology model of *Pg*DPP III and study the structure and dynamics of the ligand-free enzyme as well as of complexes with the substrates Arg_2_-2NA and Arg_2_-AMC, using computational methods.

MD simulations revealed the difference between Arg_2_-2NA and Arg_2_-AMC binding into the enzyme active site (Fig 8, S9 and S11 Figs), which is reflected in different intensity and persistence of some important hydrogen bonds (*e*.*g*. the interaction with Glu433, which according to our previous study on hDPP III takes part in the catalytic reaction [71], from the active-site motif H^432^ECLGH^437^is missing in Arg_2_-AMC-*Pg*DPP III complex; S5 Table).

Although we expected the separately expressed DPP III domain of *Pg*DPP III to have activity similar to that of the full- length protein, it showed an approximately three orders of magnitude weaker catalytic efficiency for the hydrolysis of both diarginyl arylamide substrates (Table 1). A possible explanation for the importance of the C-terminal domain for the peptidase activity is our finding that the C-terminal fragment and the DPP III lower domain motions are correlated. Our previous structural studies of human DPP III (737 amino acids) have shown that the ligand-free enzyme fluctuates between an elongated protein molecule with two domains separated by a wide cleft and more compact forms with the upper and lower lobe closer to each other [26,72]. Peptide binding boosts the closure of the binding site and shifts the protein structure to the highly compact form [62] which is prerequisite for the catalytic activity of human DPP III [71] and probably for the other members of the M49 family activity as well since they share similar protein folds [24,25]. The present MD simulations of full-length *Pg*DPP III suggest that its N-terminal DPP III region adopts a relatively compact form in solution, similar to the so called ‘semi closed form’ reported for the human orthologue (S4 Table). In addition, the simulations showed a significant reorganization in the C-terminal ARM region, that correlated with a motion of the lower DPP III domain and an increase of the compactness of the whole enzyme structure (Fig 3). Therefore, we suppose that the C-terminally appended domain influences the interdomain dynamics of the DPP III region as well as peptide binding. A comparison between the MD simulations and the experimental HDX results revealed that *Pg*DPP III adopts a more compact structure in solution than predicted by homology modeling, with the motion of the lower domain of the DPP III fragment being highly correlated with reorganization of the C-terminal ARM fragment.

The ARM-type fold is multi-helical and comprised of two curved layers of alpha helices arranged in a right-handed superhelix [73]. Domains and repeats with an ARM-like fold are found in a number of different proteins involved in various important cellular processes [74]. The ARM repeat fold was also found in the C-terminal domain of aminopeptidase B and the bifunctional enzyme leukotriene A4 hydrolase, as well as in aminopeptidase O, a human brain metallopeptidase, all members of the M1 family, which is characterized by the zinc-binding motif HEXXHX_18_E [75]. To our knowledge, the function of the ARM domain in these aminopeptidases has not been investigated in detail.

Although the complementation assays in a DNA-repair deficient *Escherichia coli* strain indicated the absence of any alkylation repair function in *Pg*DPP III, the presence of an appended ARM repeat domain at the C-terminus of *Pg*DPP III indicates multifunctionality of this protein and opens new avenues for future research.

## Supporting information

S1 Fig Multiple sequence alignment. Multiple sequence alignment of a selection of DPP III showing conservation of active site motif (M49 family zinc-binding motifs are highlighted). Multiple sequence alignment was obtained using CLUSTAL Omega with DPP III sequences for: *Homo sapiens* (Q9NY33), *Mus musculus* (Q99KK7), *Danio rerio* (Q6DI20), *Drosophila melanogaster* (Q9VHR8), *Saccharomyces cerevisiae* (Q08225), *Bacteroides thetaiotaomicron* (Q8A6N1), and *Porphyromonas gingivalis* (Q7MX92).
(DOCX)

S2 Fig 3D models of *P*.*gingivalis* DPP III. Homology modeling of the three-dimensional structure of *P*. *gingivalis* DPP III was performed using Phyre2 program. On the right AlkD and AlkF structures from *B*. *cereus*. Active site in DPP III is coloured in blue.
(DOCX)

S3 Fig HPLC-MS results. HPLC-MS results of 24 hours incubation of wild type *Pg*DPP III enzyme with ligands angiotensin II, tynorphin and IVYPW, respectively. In all three reactions there is main product detectable and small amount of side products confirming sequential cleavage of dipeptides from N-termini of substrates.
(DOCX)

S4 Fig Angiotensin II time studies. Angiotensin II time studies with *P*. *gingivalis* DPP III (A, left) and human DPP III (B, right). The same type of biotransformation occurs, although in hDPP III is faster confirming angiotensin II as very good substrate for this enzyme.
(DOCX)

S5 Fig MMS complementation assay. An aliquot of 1 μL serially diluted mid-log phase cultures of BK2118 strain transformed with pUC18PgDPP3, pUC18AlkD_like domain PgDPP3 (C-terminus), pUC18AlkD *B*. *cereus* and pUC18AlkD *P*. *gingivalis*, and with pET21a empty plasmid, was spotted on LBA plates with 2 mM MMS and incubated for 2 days at 37°C.
(DOCX)

S6 Fig The *Pg*DPP III structure obtained by comparative modelling. The DPP III part is colored orange and the ARM part yellow. Zn^2+^ is represented by gray sphere and amino acid residues that coordinate it are given in stick representation.
(DOCX)

S7 Fig Overlay of the structure obtained after 200 ns of MD simulations of the homology modelled *Pg*DPP III structure. Overlay of the structure obtained after 200 ns of MD simulations of the homology modelled *Pg*DPP III structure (magenta) and the structure obtained after 150 ns of MD simulations of the protein extracted from the simulated *Pg*DPP III- Arg_2_-2NA complex (blue). The Zn ion, represented as magenta sphere, and the amino acid residues coordinating it, represented as sticks, are encircled.
(DOCX)

S8 Fig Rgyr (Å) profile of *Pg*DPP III. Rgyr (Å) profile of *Pg*DPP III (black), its DPP IIIpart (red) and AlkD like C-terminal domain (green) determined during 200 ns long MD simulation of the *Pg*DPP III—Arg_2_-2NA complex (replica 1).
(DOCX)

S9 Fig The amino acid residues from the enzyme *Pg*DPP III binding pocket that Arg_2_-2NA molecule interacts with during 200 ns MD simulation of one replica. (TIF)

S10 Fig The electrostatic potential surface of the *Pg*DPP III binding site. The electrostatic potential surface of the *Pg*DPP III binding site for the structure of the *Pg*DPP III—Arg_2_-2NA complex obtained after 150 ns of MD simulations (red and blue surface represent position of the negativelly and positively charged residues, respectively) Substrate, Arg_2_-2NA, is shown in stick representation.
(DOCX)

S11 Fig Stabilization of Arg_2_-AMC in the *Pg*DPP III active site. Structure obtained after 150 ns of MD simulations. Zn^2+^ is represented as a gray sphere.
(DOCX)

S12 Fig Correlation of the HDX results with the theoretical results based on the MD simulations of the inital (homology modelled) *Pg*DPP III structure. The structure is obtained during 200 ns of MD simulation (50 ns equilibration + 150 productive MD).
(DOCX)

S13 Fig Correlation of the HDX and MD results. Correlation of the HDX results with the theoretical results based on the MD simulations of the *Pg*DPP III structure extracted from the simulated *Pg*DPP III—Arg_2_-2NA complex and simulated for 100 ns.
(DOCX)

S14 Fig Correlation of the HDX and MD results. Correlation of the HDX results with the theoretical results based on the MD simulations of the inital (homology modelled) *Pg*DPP III structure during 200 ns of MD simulation (50 ns equilibration + 150 productive MD) for the DPP III part of the structure (amino acids 14–654).
(DOCX)

S1 Table Primers. (DOCX)

S2 Table Substrate specificity for *Pg*DPP III. (DOCX)

S3 Table Thermodynamic parameters of ITC experiments with angiotensin II, tynorphin and IVYPW. The experiments were made in duplicate.
(DOCX)

S4 Table The secondary structure composition of the DPP III and ARM regions. (DOCX)

S5 Table Populations (% of the frames sampled during MD simulations) of the selected strong intermolecular hydrogen bonds. (DOCX)
