# Supplementary figures and images for: A novel Porphyromonas gingivalis enzyme: An atypical dipeptidyl peptidase III with an ARM repeat domain
Source: PLoS One. 2017 Nov 30;12(11):e0188915. doi: 10.1371/journal.pone.0188915 (PMC5708649; doi:10.1371/journal.pone.0188915)

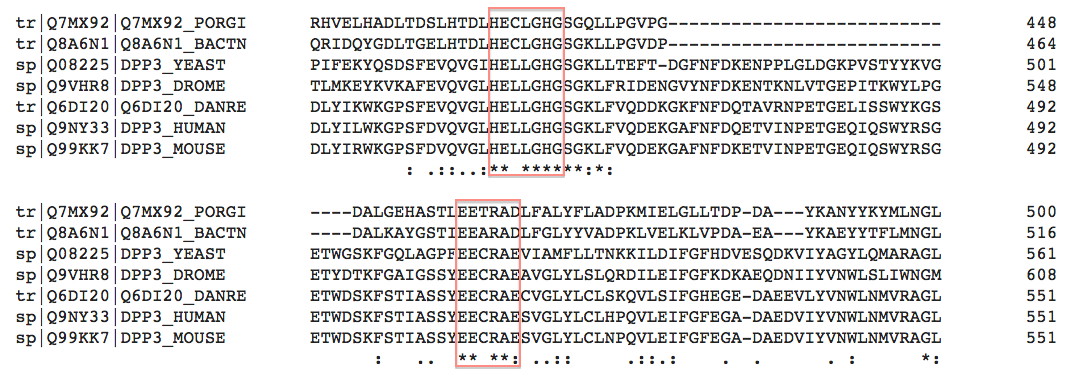

Supplement: S1 Fig — Multiple sequence alignment of a selection of DPP III showing conservation of active site motif (M49 family zinc-binding motifs are highlighted). Multiple sequence alignment was obtained using CLUSTAL Omega with DPP III sequences for: Homo sapiens (Q9NY33), Mus musculus (Q99KK7), Danio rerio (Q6DI20), Drosophila melanogaster (Q9VHR8), Saccharomyces cerevisiae (Q08225), Bacteroides thetaiotaomicron (Q8A6N1), and Porphyromonas gingivalis (Q7MX92). (DOCX) [file pone.0188915.s001.docx]

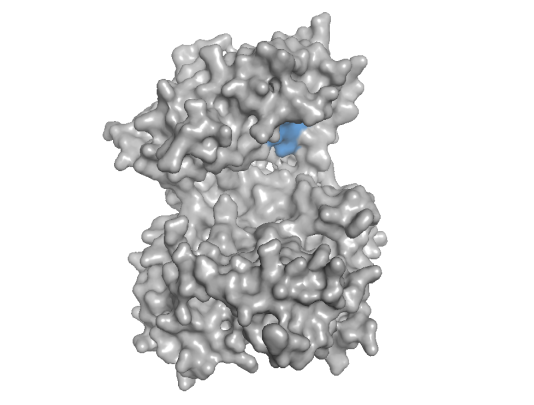

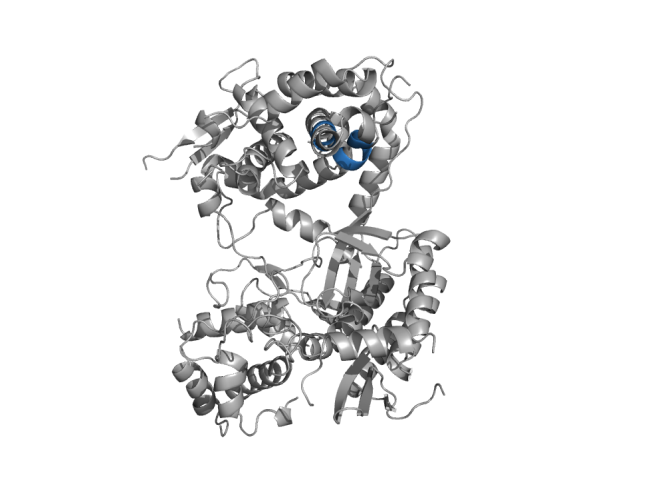


**DPP III domain**

**(1-663)**

**AlkD/F like domain**

**(664-886)**


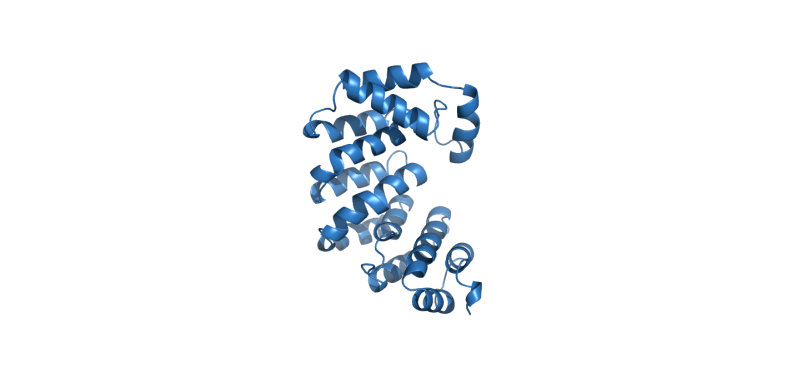

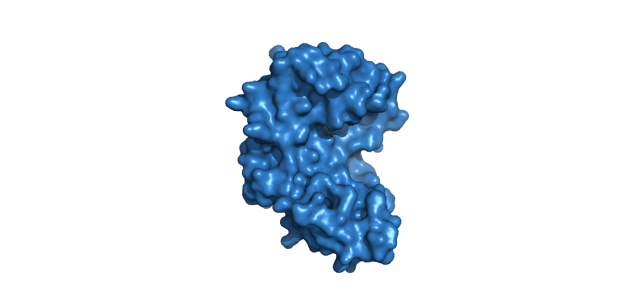

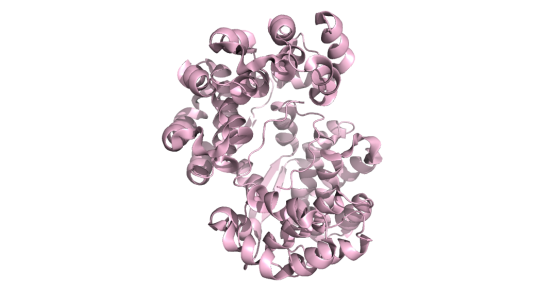

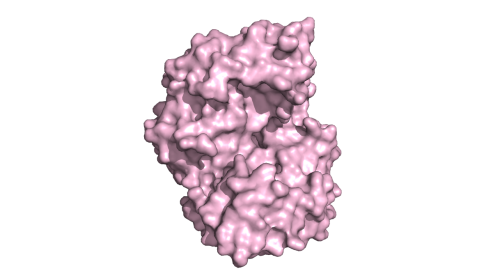


**AlkD *B. cereus***

**(pdb: 3BVS)**

**AlkF *B. cereus***

**(pdb: 3ZBO)**


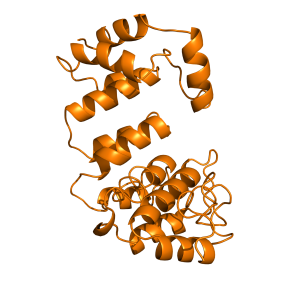

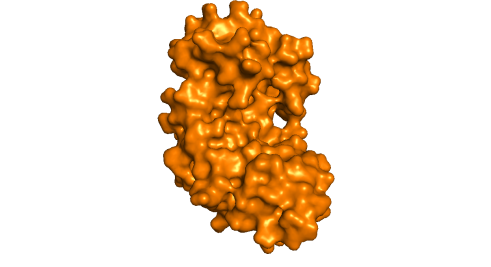

Supplement: S2 Fig — Homology modeling of the three-dimensional structure of P. gingivalis DPP III was performed using Phyre2 program. On the right AlkD and AlkF structures from B. cereus. Active site in DPP III is coloured in blue. (DOCX) [file pone.0188915.s002.docx]

**
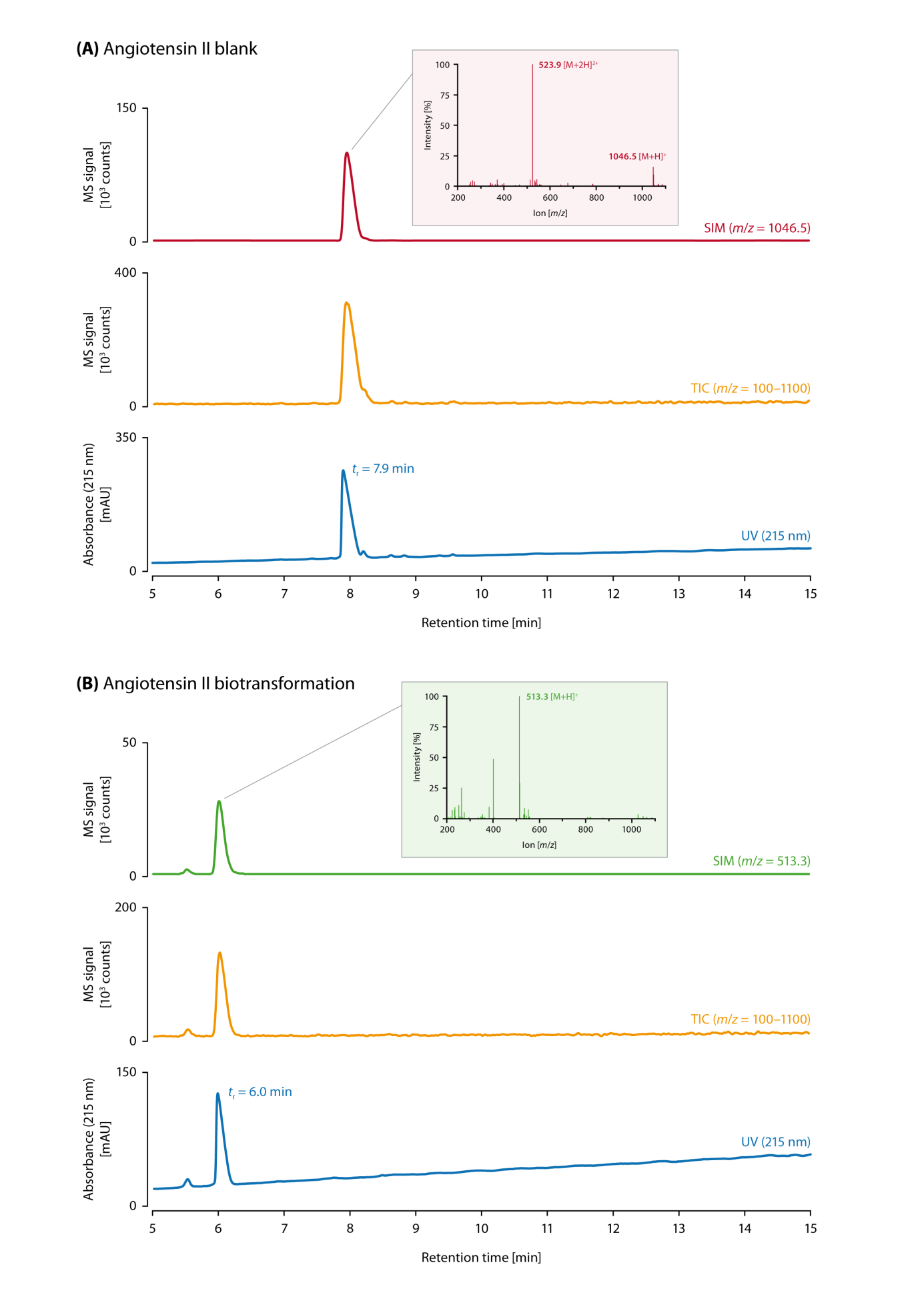
**

**
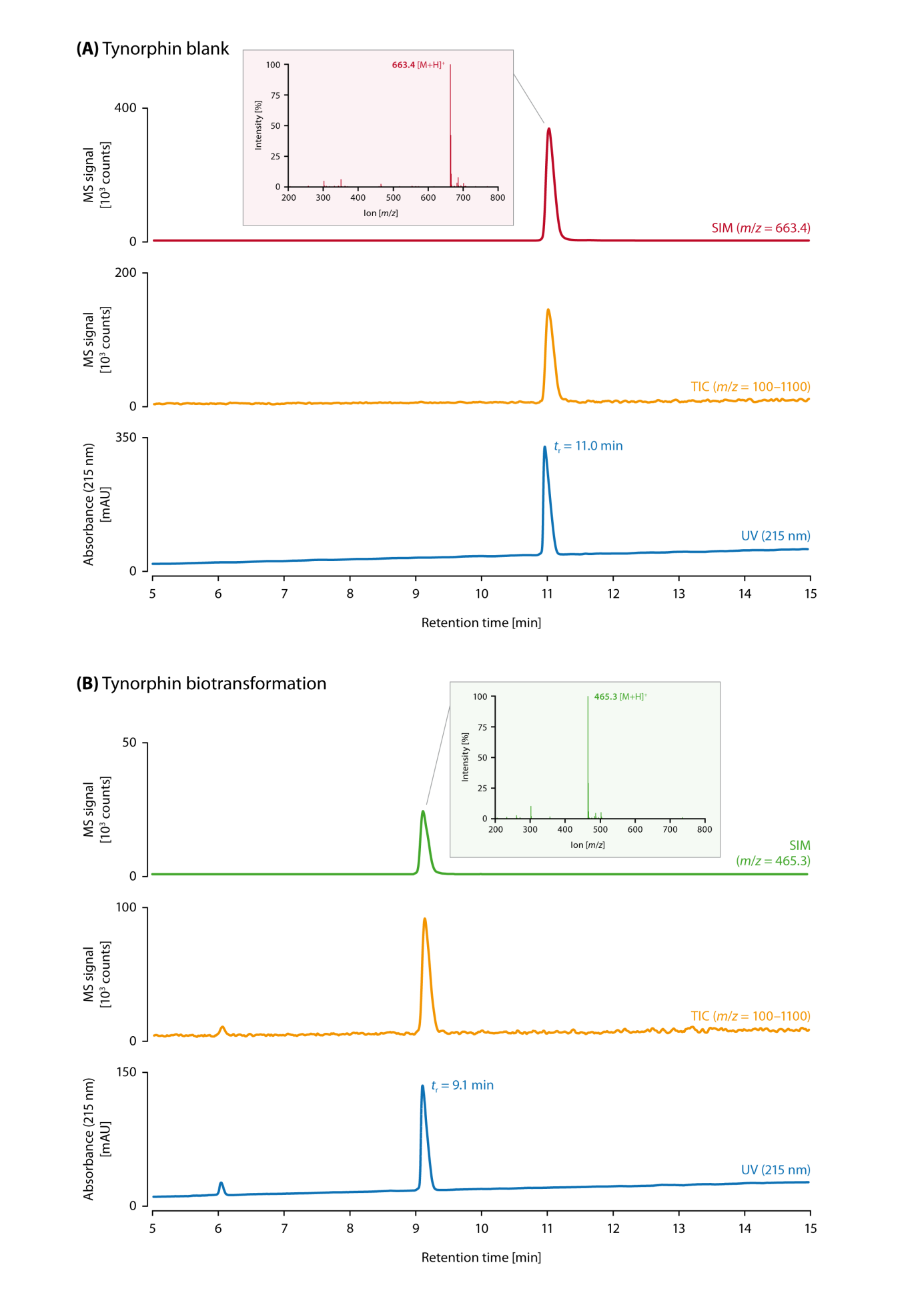
**

**
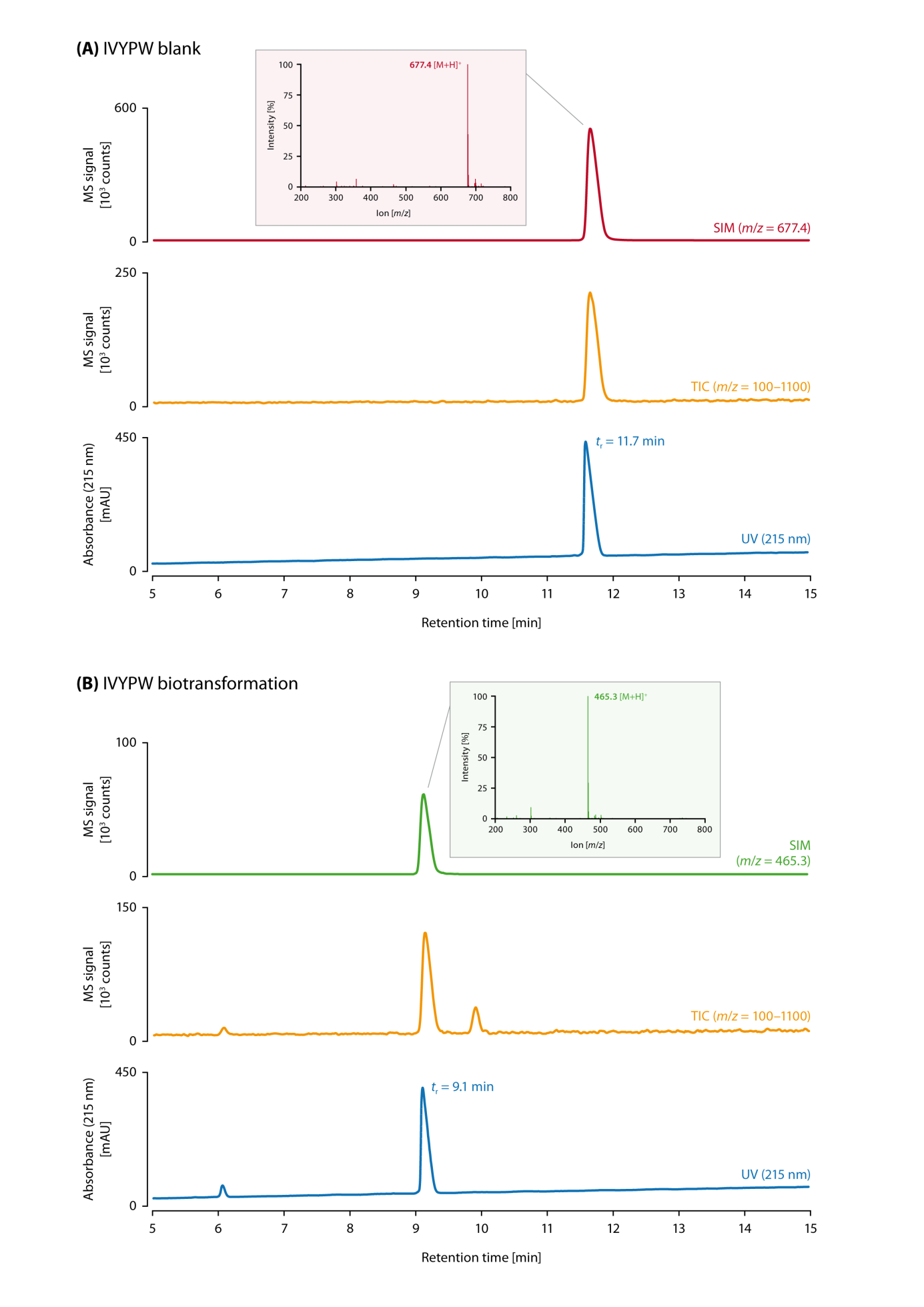
**

Supplement: S3 Fig — HPLC-MS results of 24 hours incubation of wild type PgDPP III enzyme with ligands angiotensin II, tynorphin and IVYPW, respectively. In all three reactions there is main product detectable and small amount of side products confirming sequential cleavage of dipeptides from N-termini of substrates. (DOCX) [file pone.0188915.s003.docx]

**
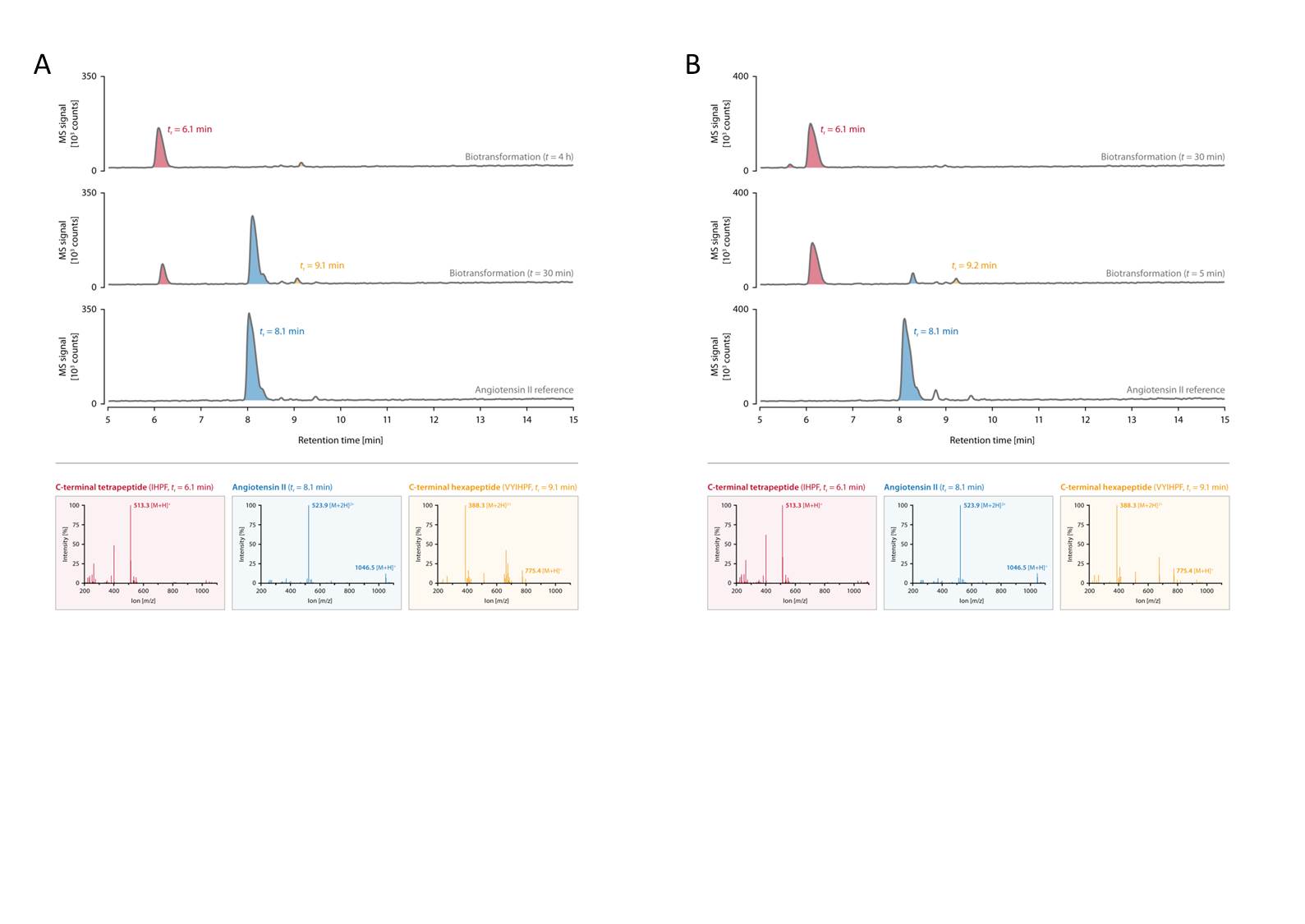
**

Supplement: S4 Fig — Angiotensin II time studies with P. gingivalis DPP III (A, left) and human DPP III (B, right). The same type of biotransformation occurs, although in hDPP III is faster confirming angiotensin II as very good substrate for this enzyme. (DOCX) [file pone.0188915.s004.docx]

Cell number

AlkD *P. gingivalis*

empty plasmid

*Pg*DPP III

AlkD_like domain *Pg*DPP III

AlkD *B.cereus*


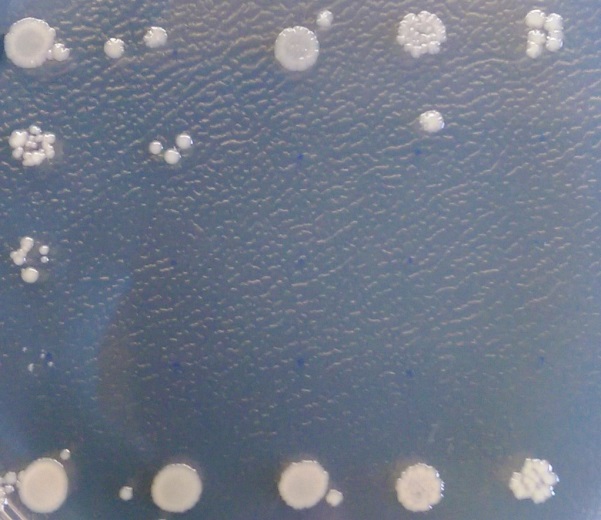

Supplement: S5 Fig — An aliquot of 1 μL serially diluted mid-log phase cultures of BK2118 strain transformed with pUC18PgDPP3, pUC18AlkD_like domain PgDPP3 (C-terminus), pUC18AlkD B. cereus and pUC18AlkD P. gingivalis, and with pET21a empty plasmid, was spotted on LBA plates with 2 mM MMS and incubated for 2 days at 37°C. (DOCX) [file pone.0188915.s005.docx]

**
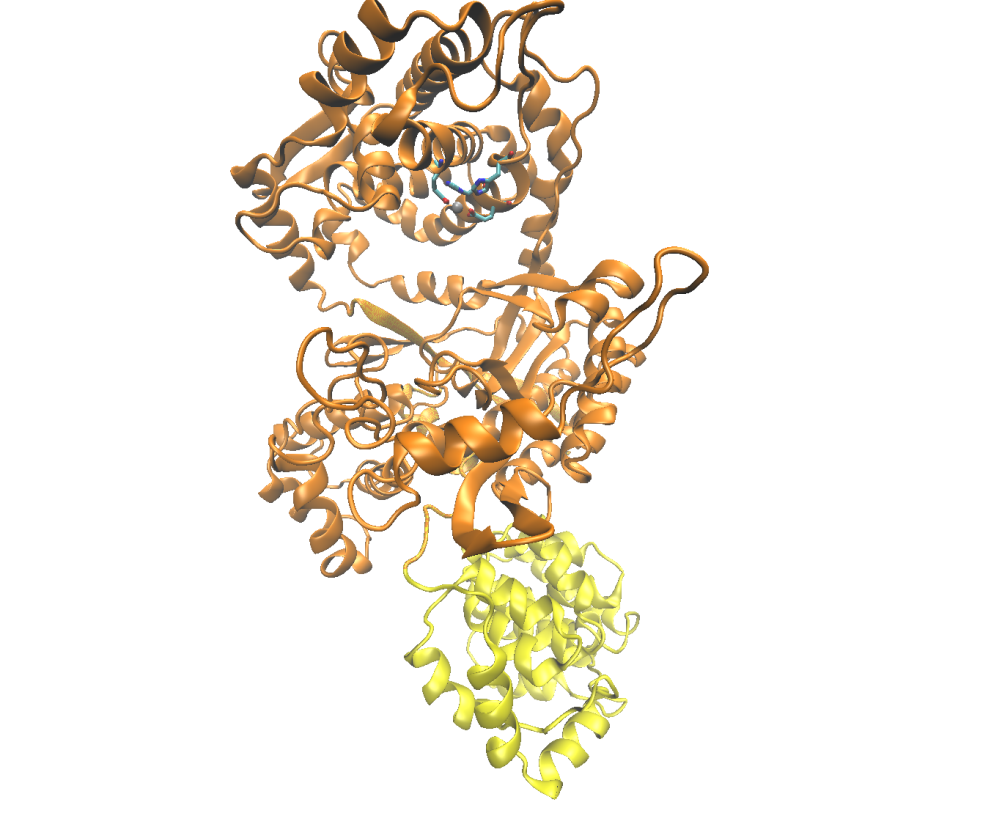
**

Supplement: S6 Fig — The DPP III part is colored orange and the ARM part yellow. Zn2+ is represented by gray sphere and amino acid residues that coordinate it are given in stick representation. (DOCX) [file pone.0188915.s006.docx]

**
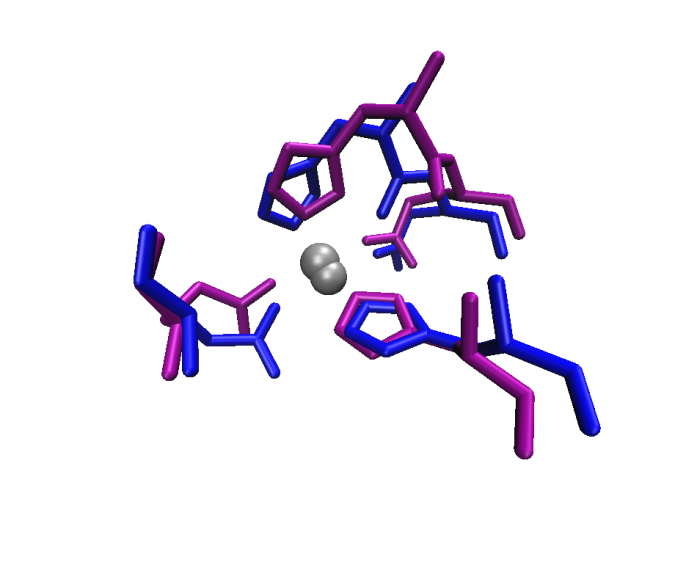

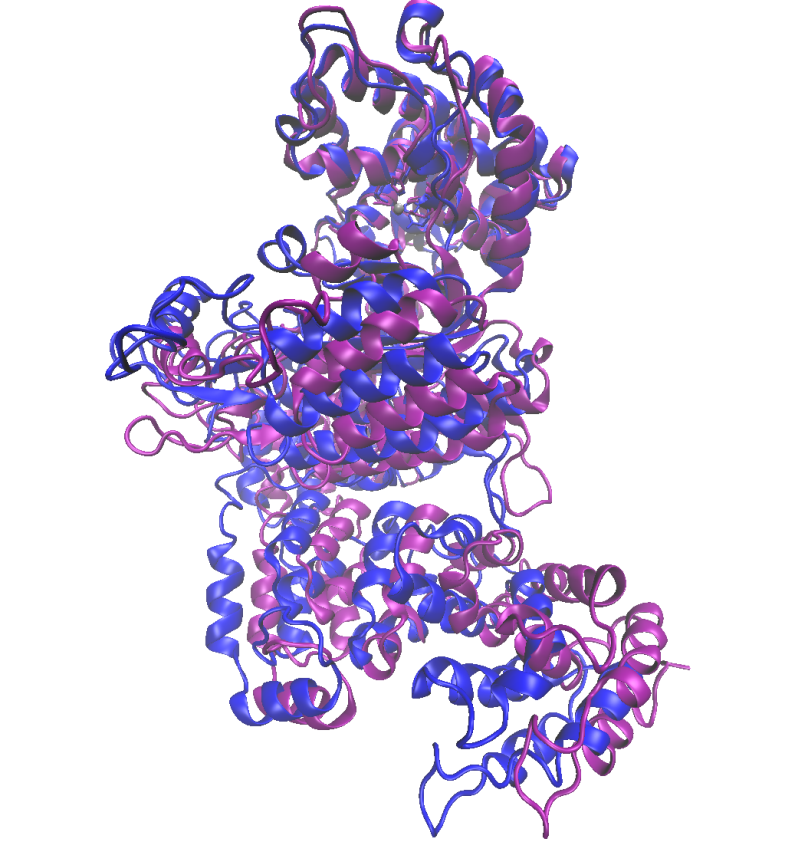
**

Supplement: S7 Fig — Overlay of the structure obtained after 200 ns of MD simulations of the homology modelled PgDPP III structure (magenta) and the structure obtained after 150 ns of MD simulations of the protein extracted from the simulated PgDPP III- Arg2-2NA complex (blue). The Zn ion, represented as magenta sphere, and the amino acid residues coordinating it, represented as sticks, are encircled. (DOCX) [file pone.0188915.s007.docx]

**
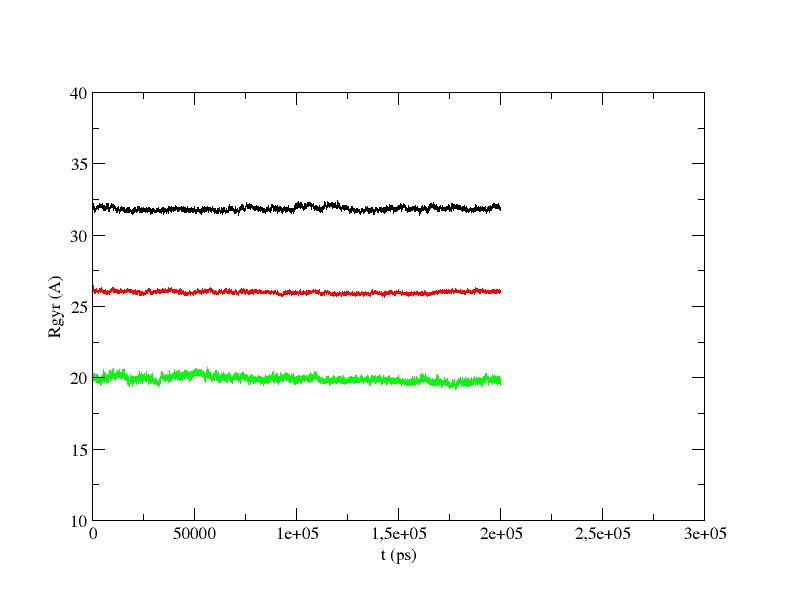
**

Supplement: S8 Fig — Rgyr (Å) profile of PgDPP III (black), its DPP IIIpart (red) and AlkD like C-terminal domain (green) determined during 200 ns long MD simulation of the PgDPP III—Arg2-2NA complex (replica 1). (DOCX) [file pone.0188915.s008.docx]

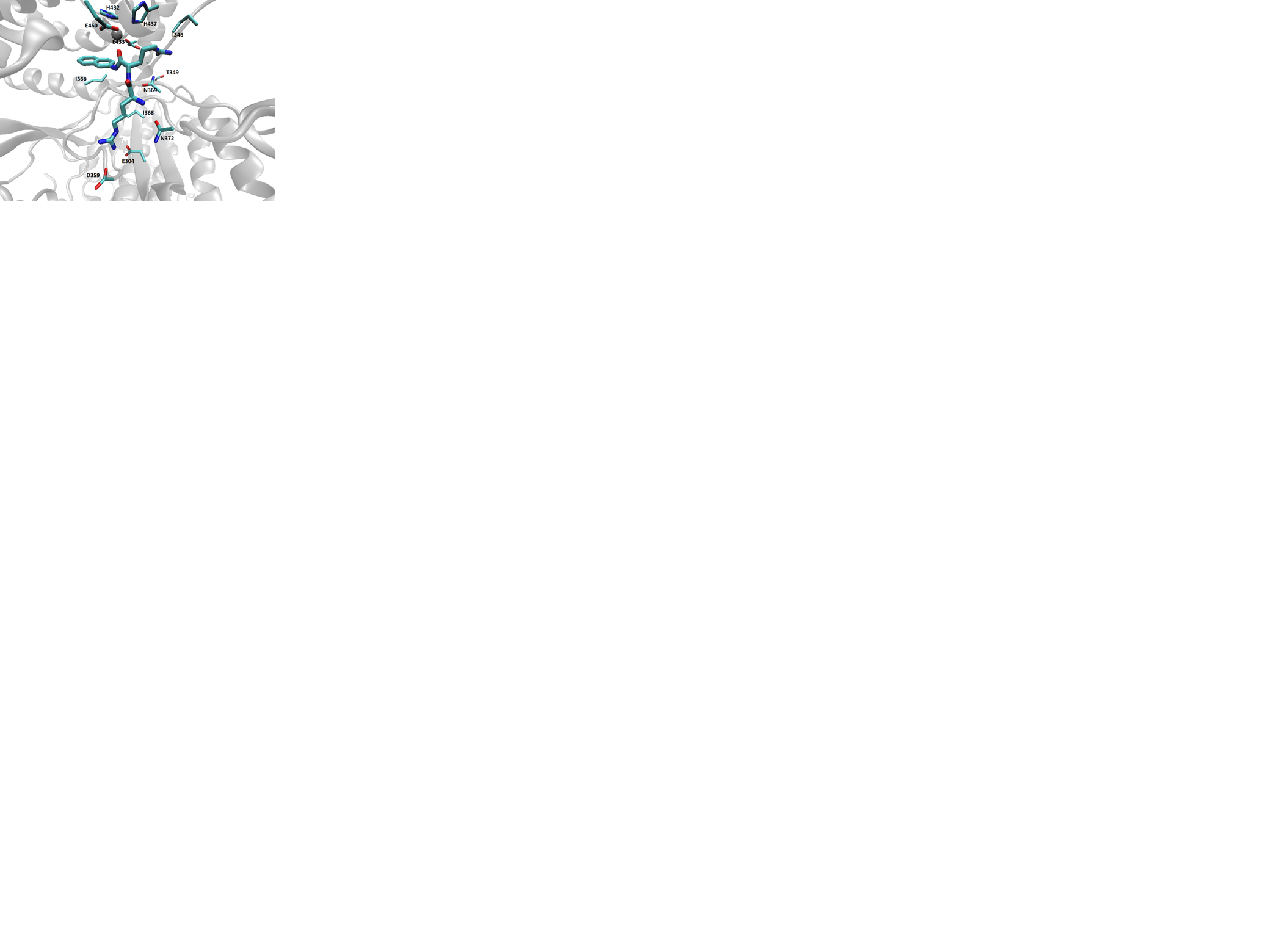

Supplement: S9 Fig — (TIF) [file pone.0188915.s009.tif]

**
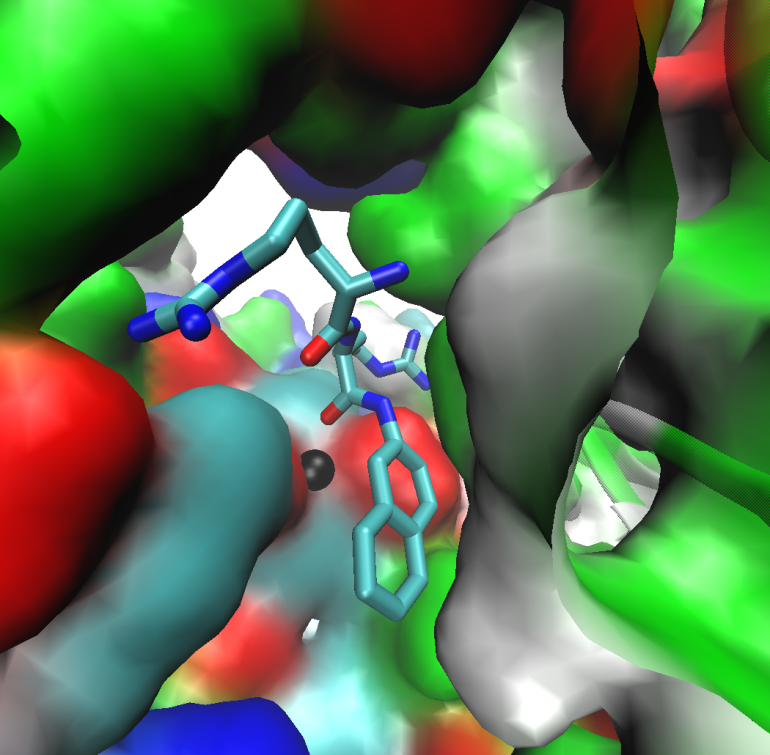
**

Supplement: S10 Fig — The electrostatic potential surface of the PgDPP III binding site for the structure of the PgDPP III—Arg2-2NA complex obtained after 150 ns of MD simulations (red and blue surface represent position of the negativelly and positively charged residues, respectively) Substrate, Arg2-2NA, is shown in stick representation. (DOCX) [file pone.0188915.s010.docx]

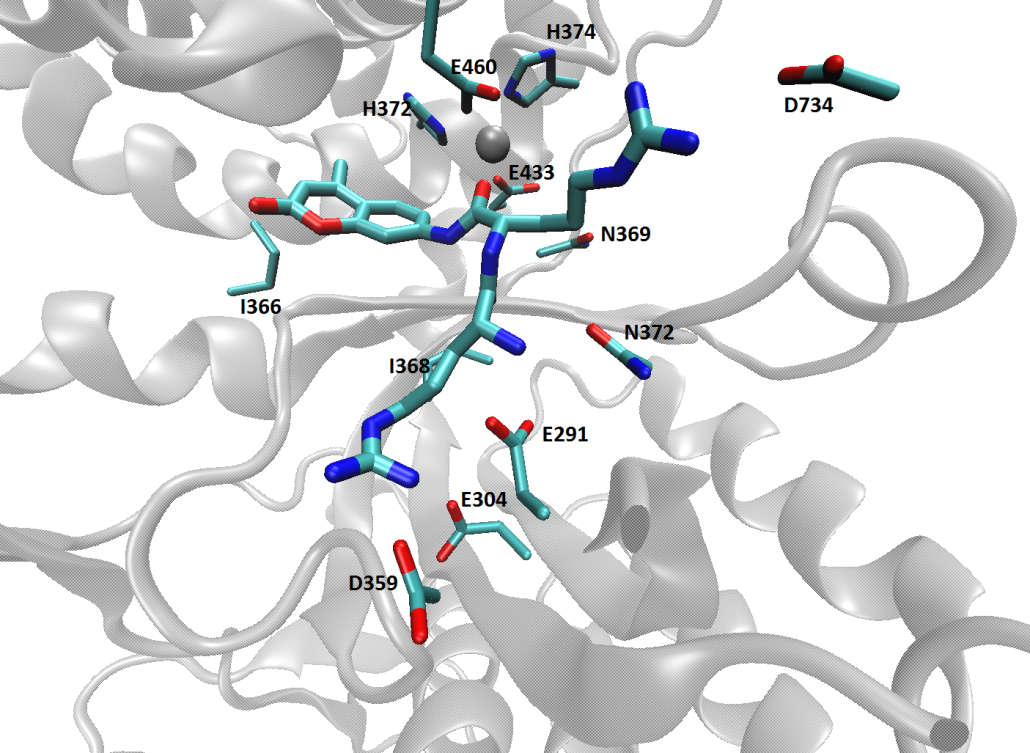

Supplement: S11 Fig — Structure obtained after 150 ns of MD simulations. Zn2+ is represented as a gray sphere. (DOCX) [file pone.0188915.s011.docx]

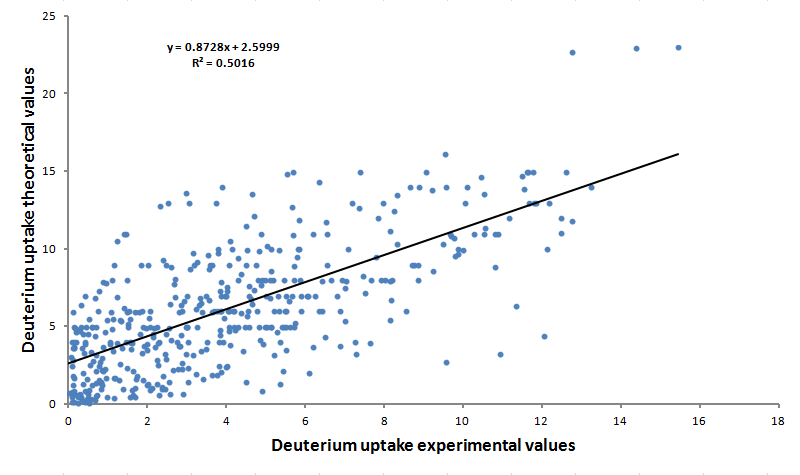

Supplement: S12 Fig — The structure is obtained during 200 ns of MD simulation (50 ns equilibration + 150 productive MD). (DOCX) [file pone.0188915.s012.docx]

**
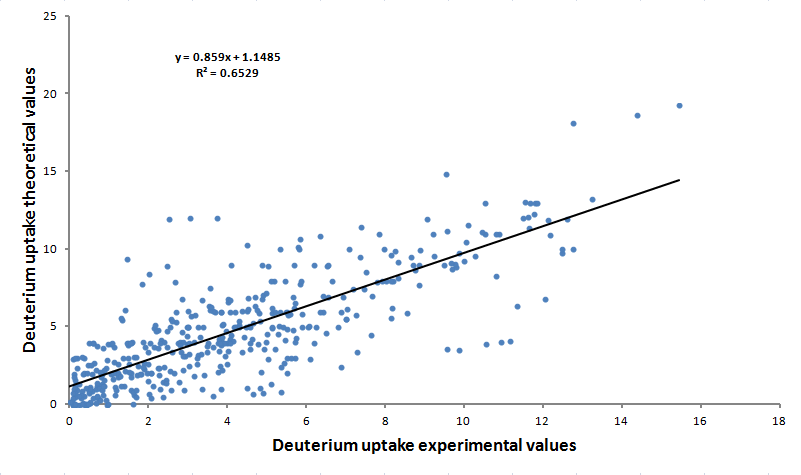
**

Supplement: S13 Fig — Correlation of the HDX results with the theoretical results based on the MD simulations of the PgDPP III structure extracted from the simulated PgDPP III—Arg2-2NA complex and simulated for 100 ns. (DOCX) [file pone.0188915.s013.docx]

**
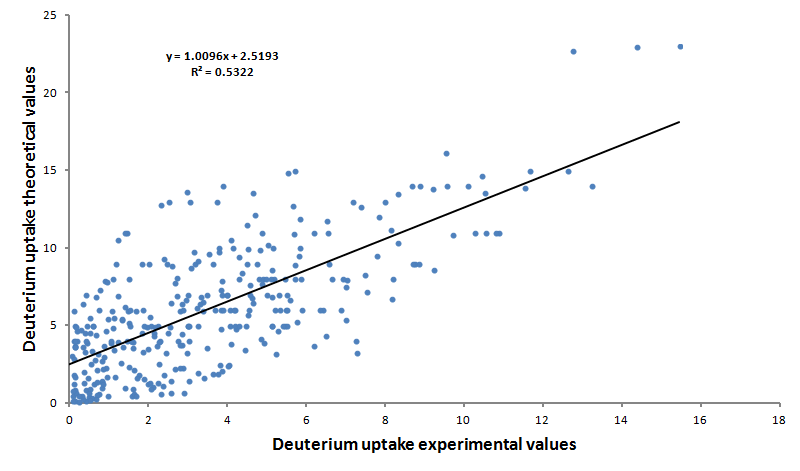
**

Supplement: S14 Fig — Correlation of the HDX results with the theoretical results based on the MD simulations of the inital (homology modelled) PgDPP III structure during 200 ns of MD simulation (50 ns equilibration + 150 productive MD) for the DPP III part of the structure (amino acids 14–654). (DOCX) [file pone.0188915.s014.docx]
