# Supplementary material for: A novel Porphyromonas gingivalis enzyme: An atypical dipeptidyl peptidase III with an ARM repeat domain
Source: PLoS One. 2017 Nov 30;12(11):e0188915. doi: 10.1371/journal.pone.0188915 (PMC5708649; doi:10.1371/journal.pone.0188915)
Supplement: S1 Table — (DOCX) [file pone.0188915.s015.docx]

**S1 Table. Primers.**

Primers used for the expression of full-lenght and DPP III fragments, and for cloning to pUC18 plasmid for MMS complementation assay. Restriction sites are underlined. Start codon in primers for pLATE31/LIC cloning are bolded, and complementary parts of primers are in italic. The underlined and bolded nucleotides represent the mutated codon.

| Name | Sequence (5'-3') |
| --- | --- |
| *Pg*DPP3_(1-886)_*Nhe*I | CGAGGAGCTAGCATGACAAAAGAAACAACCCAACACCG |
| *Pg*DPP3_(1-886)_*Xho*I | CCGCTCGAGCTCGTCCTTTGGGTAGAAGTCGAGTTC |
| *Pg*DPP3_(1-679)_fwd | AGAAGGAGATATAACT**ATG***ACAAAAGAAACAACCCAACACC* |
| *Pg*DPP3_(1-679)_rev | GTGGTGGTGATGGTGATGGCC*TAGTACACCGTCCATCGCCCGTCG* |
| *Pg*DPP3_(648-886)_AlkDlike1_fwd | GTGGTGGTGATGGTG**ATG**GCC*CTCGTCCTTTGGGTAGAAGTCGAG* |
| *Pg*DPP3_(660-886)_AlkDlike2_fwd | AGAAGGAGATATAACT**ATG***CTTTTGCAGGAGGCACGAAGATTG* |
| *Pg*DPP3_(675-886)_AlkDlike3_fwd | AGAAGGAGATATAACT**ATG**GACGGTGTACTATCGGCCAG |
| *Pg*DPP3___AlkDlike1_rev | GTGGTGGTGATGGTGATGGCC*CTCGTCCTTTGGGTAGAAGTCGAG* |
| pUC18_*Pg*DPP3_(1-886)_*EcoR*I | CCGGAATTC*GATGACAAAAGAAACAACCCAACACCG* |
| pUC18_*Pg*DPP3_(1-886)_*Pst*I | TTTTCTGCAGTTA*CTCGTCCTTTGGGTAGAAGTCGAG* |
| pUC18_*Pg*DPP3_(648-886)_*EcoR*I | CCGGAATTCGATG*GCAGAATATAGCTTCCTGCCTACAGAC* |
| pUC18_*Pg*AlkD_*EcoR*I | CCGGAATTC*GATGGATCATAGCCAACTGACCGC* |
| pUC18_*Pg*AlkD_*Pst*I | TTTTCTGCAGTTA*TTTCCCTTTGGTCAGGTAGTAAGAAG* |
| E433A_*Pg*DPP3 | CATGTCCGAGGCATGCGT**GCA**GGTCGGTG |

648-879: 660-879; 675-879 aa fragments
