# Supplementary material for: A novel Porphyromonas gingivalis enzyme: An atypical dipeptidyl peptidase III with an ARM repeat domain
Source: PLoS One. 2017 Nov 30;12(11):e0188915. doi: 10.1371/journal.pone.0188915 (PMC5708649; doi:10.1371/journal.pone.0188915)
Supplement: S2 Table — (DOCX) [file pone.0188915.s016.docx]

**S2 Table. Substrate specificity for *Pg*DPP III.**

| Substrate | Relative hydrolysis rate (%) |
| --- | --- |
| **Arg-Arg-2NA** | **100** |
| **Phe-Arg-2NA** | **68.11** |
| **Ala-Arg-2NA** | **60.84** |
| **Gly-Arg-2NA** | **22.87** |
| **Lys-Ala-2NA** | **17.96** |
| **Ala-Ala-2NA** | **16.09** |
| **Pro-Arg-2NA** | **2.63** |
| His-Phe-2NA | 1.74 |
| His-Ser-2NA | 1.82 |
| Glu-His-2NA | 1.45 |
| Gly-Phe-2NA | 1.39 |
| Asp-Arg-2NA | 1.19 |
| BANA | 0 |
