# Supplementary material for: A novel Porphyromonas gingivalis enzyme: An atypical dipeptidyl peptidase III with an ARM repeat domain
Source: PLoS One. 2017 Nov 30;12(11):e0188915. doi: 10.1371/journal.pone.0188915 (PMC5708649; doi:10.1371/journal.pone.0188915)
Supplement: S3 Table — The experiments were made in duplicate. (DOCX) [file pone.0188915.s017.docx]

**S3** **Table. Thermodynamic parameters of ITC experiments with angiotensin II, tynorphin and IVYPW.** The experiments were made in duplicate.

| Thermodynamic parameters | Angiotensin-II | Tynorphin | IVYPW |
| --- | --- | --- | --- |
| *K*_d_ (µM) | 0.90 ± 0.12 | 7.30 ± 0.83 | 9.35 ± 0.73 |
| *ΔH* (kJ.mol^-1^) | -9.38 ± 0.12 | 23.58 ± 0.72 | 35.82 ± 0.90 |
| *ΔG* (kJ.mol^-1^) | -34.51 ± 0.34 | -29.32 ± 0.28 | -28.71 ± 0.19 |
| *-TΔS* (kJ.mol^-1^) | -25.13 ± 0.36 | -52.90 ± 0.78 | -64.53 ± 0.92 |
