# Supplementary material for: A novel Porphyromonas gingivalis enzyme: An atypical dipeptidyl peptidase III with an ARM repeat domain
Source: PLoS One. 2017 Nov 30;12(11):e0188915. doi: 10.1371/journal.pone.0188915 (PMC5708649; doi:10.1371/journal.pone.0188915)
Supplement: S4 Table — (DOCX) [file pone.0188915.s018.docx]

**S4 Table. The secondary structure composition of the DPP III and ARM regions**. Values in the initial, by homology modeling derived structure and during the 200 ns and 150 ns long MD simulations of the ligand free protein.

| *Pg*DPP III structure | region | The secondary structure elements (%) | | | |
| --- | --- | --- | --- | --- | --- |
|  |  | β strands | helical | turns | bends |
| Homology model | DPP III | 10.3 | 48.6 | 10.5 | 9.9 |
|  | ARM | 7.7 | 30.8 | 7.7 | 19.2 |
| Simulated 200 ns | DPP III | 9.6 | 46.0 | 12.60 | 10.3 |
|  | ARM | 0.0 | 62.7 | 11.6 | 7.1 |
| Simulated 150 ns | DPP III | 11.1 | 48.3 | 12.90 | 9.5 |
|  | ARM | 0.0 | 59.1 | 13.3 | 9.8 |
