# Supplementary material for: A novel Porphyromonas gingivalis enzyme: An atypical dipeptidyl peptidase III with an ARM repeat domain
Source: PLoS One. 2017 Nov 30;12(11):e0188915. doi: 10.1371/journal.pone.0188915 (PMC5708649; doi:10.1371/journal.pone.0188915)
Supplement: S5 Table — (DOCX) [file pone.0188915.s019.docx]

**S5 Table. Populations (% of the frames sampled during MD simulations) of the selected strong intermolecular hydrogen bonds**. Values averaged over 200 and 150 ns of MD simulations of the *Pg*DPP III – Arg_2_-2NA complex (replica1 and replica2, respectively) and the *Pg*DPP III - Arg_2_-AMC complex. The hydrogen bonds were calculated with CPPTRAJ using default parameters. Only the bonds with population above 10% (at least for one complex) during the simulations are listed.

|  | ***Pg*DPP III – Arg_2_-2NA - Replica1** | ***Pg*DPP III – Arg_2_-2NA – Replica2** | ***Pg*DPP III - Arg_2_-AMC** |
| --- | --- | --- | --- |
| Asp359 | 100 | 57 | 100 |
| Asn369 | 75 | 86 | 31 |
| Glu304 | 69 | 49 | 97 |
| Glu433 | 57 | 63 | - |
| Gly367 | 18 | 58 | 12 |
| Ser347 | 15 | 4 | - |
| Ser360 | 12 | 7 | 4 |
| Ser384 | 10 | 6 | - |
| Glu291 | 6 | 69 | 100 |
| Asn372 | 6 | 3 | 100 |
| Glu460 | - | 1 | 41 |
| Asp374 | - | - | 51 |
